# Identification of DNA-Damage DNA-Binding Protein 1 as a Conditional Essential Factor for Cytomegalovirus Replication in Interferon-γ-Stimulated Cells

**DOI:** 10.1371/journal.ppat.1002069

**Published:** 2011-06-16

**Authors:** Mirko Trilling, Vu Thuy Khanh Le, Manuela Fiedler, Albert Zimmermann, Elke Bleifuß, Hartmut Hengel

**Affiliations:** Institute for Virology, Heinrich-Heine-University Düsseldorf, Düsseldorf, Germany; Oregon Health & Science University, United States of America

## Abstract

The mouse cytomegaloviral (MCMV) protein pM27 represents an indispensable factor for viral fitness *in vivo* selectively, antagonizing *signal transducer and activator of transcription 2* (STAT2)-mediated interferon signal transduction. We wished to explore by which molecular mechanism pM27 accomplishes this effect. We demonstrate that pM27 is essential and sufficient to curtail the protein half-life of STAT2 molecules. Pharmacologic inhibition of the proteasome restored STAT2 amounts, leading to poly-ubiquitin-conjugated STAT2 forms. PM27 was found in complexes with an essential host ubiquitin ligase complex adaptor protein, *DNA-damage DNA-binding protein* (DDB) 1. Truncation mutants of pM27 showed a strict correlation between DDB1 interaction and their ability to degrade STAT2. SiRNA-mediated knock-down of DDB1 restored STAT2 in the presence of pM27 and strongly impaired viral replication in interferon conditioned cells, thus phenocopying the growth attenuation of *M27*-deficient virus. In a constructive process, pM27 recruits DDB1 to exploit ubiquitin ligase complexes catalyzing the obstruction of the STAT2-dependent antiviral state of cells to permit viral replication.

## Author Summary

Cytomegaloviruses are strictly species-specific. Mouse cytomegalovirus (MCMV) is a prototypical β-herpesvirus, infecting *Mus musculus* as natural host and is closely related to the human pathogenic cytomegalovirus (HCMV, HHV-5) which both establish lifelong infection. Thus, MCMV infection constitutes an important model for HCMV pathogenesis. Cytomegaloviral evasion from innate immunity has been observed in many respects, but the molecular mechanisms of most viral factors are still elusive. We recently identified the MCMV-encoded protein pM27 to be required for efficient viral replication in the presence of interferons *in vitro* and to be essential *in vivo*. We identified STAT2, a mediator of interferon signalling, as target of pM27. Here we identify the cellular machinery exploited by pM27 to reduce the STAT2 protein half-life. PM27 was sufficient to induce poly-ubiquitination of STAT2, tagging it for proteasomal degradation. Since pM27 lacks domains found within ubiquitin-ligases, we conducted a search for cellular co-factors. We found DDB1, an essential cellular ubiquitin-ligase complex adaptor protein, to associate with pM27. Ablation of DDB1 increased viral susceptibility towards interferon, phenocopying the attenuation of ΔM27-MCMV. This defines DDB1 as conditional essential host factor of CMV replication. Our findings exemplify how cytomegaloviruses exploit an essential host protein to circumvent innate immunity.

## Introduction

Cytomegaloviruses (CMVs) constitute prototypical β-herpesviruses. 50–95% of the global adult population are infected lifelong with human CMV (HCMV). HCMV is a leading cause of disease burden of newborns in western countries due to transplacental transmission of the virus from the mother to the foetus during pregnancy [1]. HCMV infections can also cause life-threatening symptoms in immunocompromised individuals. As a result of an intimately shared evolutionary history with their hosts, CMVs are highly species-specific precluding *in vivo* analysis of HCMV in small animal models, hampering our understanding of HCMV pathogenesis. Infection of mice with mouse cytomegalovirus (MCMV) has been proven to be a suitable model to study CMV pathogenesis [2].

A coordinated response of interferons (IFNs) together with T- and NK-cells controls MCMV reactivation from latency *in vivo* [3]. Consistently, cells with deficiencies in either the IFN induction or the IFN signalling system show increased MCMV susceptibility [4]–[9] underscoring the indispensable role of both type I (IFN-α/β) as well as type II (IFN-γ) IFN for the control of CMV replication.

IFNs directly trigger immune responses by inducing antiviral effector mechanisms and indirectly by activating adaptive immune responses. Thereby, IFNs constitute a constant and selecting pressure for CMV, highlighted by the multitude of viral IFN antagonists [10]. IFNs elicit their antiviral activity by initiating specific transcriptional programs. Upon binding of type I IFNs to the cognate receptor, the Janus kinase (Jak)-signal transducer and activator of transcription (STAT) signalling cascade is activated. Jak1 and tyrosine kinase 2 initiate a phosphorylation cascade at the IFN receptor chain 2 and 1, respectively. The Janus kinases phosphorylate STAT1 and STAT2. Phosphorylated STATs dimerize due to a reciprocal SH2-phospho-Tyr-interaction. The STAT heterodimers, together with the IFN regulatory factor 9 (IRF-9), constitute the IFN stimulated gene factor 3 (ISGF3), which translocates to the nucleus, binds to IFN stimulated response elements (ISRE) of IFN-inducible genes (ISGs) and recruits the transcriptional machinery to express the respective gene.

We identified the protein pM27 as MCMV-encoded inhibitor of the Jak-STAT signalling cascade [11]. *M27* is an *early-late* expressed gene essential for reducing STAT2 amounts upon MCMV infection. ΔM27-MCMV replication is attenuated upon IFN treatment *in vitro*, reproducing the observed attenuation *in vivo* [11], [12]. Interestingly, ΔM27-MCMV shows a remarkable growth reduction in IFN-γ-treated cells, revealing the importance of an IFNAR1-independent IFNGR1-initiated activation of STAT2 [11]. ΔM27-MCMV induces increased levels of ISGs [13] but does not induce more IFN-β mRNA [14], consistent with the notion that MCMV antagonizes IFN-β enhanceosome assembly *M27*-independently before an ISRE-dependent positive feed-back loop can be initiated [14].

The present study aimed to delineate the molecular mechanism and to identify host factors exploited by pM27. Here we report that pM27 exploits *DNA-damage DNA-binding protein* (DDB)1-dependent ubiquitin (Ub)-ligase complexes to catalyze ubiquitin-conjugation of STAT2. Ablation of host DDB1 phenocopied genetic deletion of *M27* from the viral genome, demonstrating that viral fitness relies on the availability of a distinct host factor, DDB1.

## Results

### The pM27-dependent reduction of STAT2 occurs post-transcriptionally

Having demonstrated that pM27 is essential and sufficient to decrease STAT2 amounts and that both proteins co-precipitate [11], we intended to elucidate the mechanism of pM27. MCMV mutants expressing C-terminal HA-epitope tagged pM27 (M27-HA-MCMV) or pM28 (M28-HA-MCMV), the gene product of the *M28* gene directly adjacent to *M27* in the MCMV genome, were proven to be able to reduce STAT2, whereas ΔM27-MCMV and UV-inactivated virus did not decrease STAT2 amounts (Figure S1), indicating suitability of above mentioned mutants for further analysis. A quantitative experimental setup reveals a time-dependent decline of endogenous STAT2 amounts upon infection with wt-MCMV but not upon infection with ΔM27-MCMV until 24 h post infection (Figure S2). Pre-incubation with IFN-γ significantly increased levels of STAT2 but did not comprise pM27 function (Figure S3). During the early phase (24 h post infection) of MCMV replication pM27 seems to be the only MCMV-encoded protein significantly reducing STAT2 amounts (Figure S1, S2 and S5). Nevertheless, at late times of replication (≥48 h post infection) some STAT2 reduction was observed in ΔM27-MCMV infected cells, raising the possibility that additional MCMV gene products might affect STAT2 (Figure S3).

**Figure 1:**
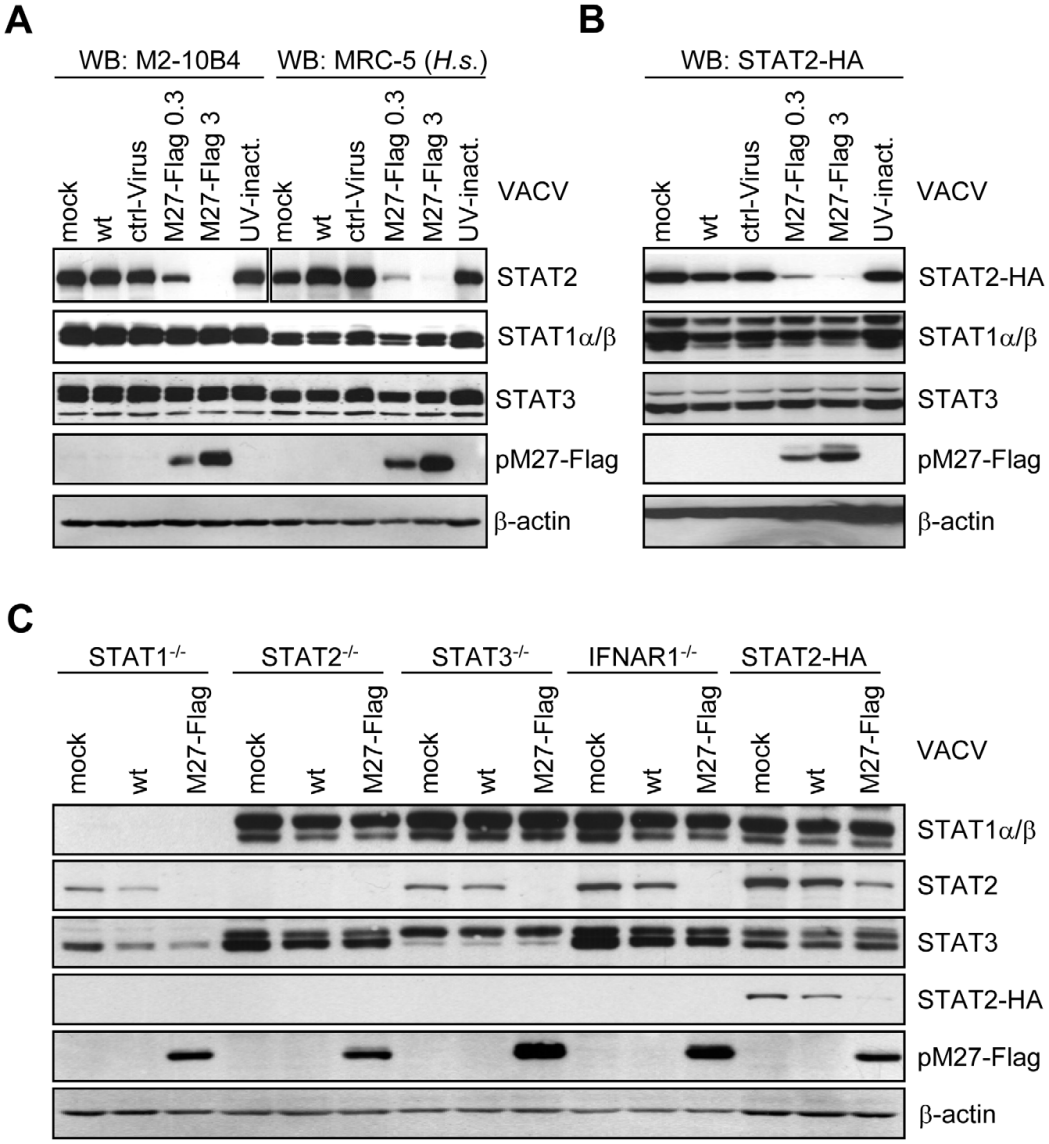
pM27 is essential and sufficient to reduce the amount of human and mouse STAT2. (**A**) Human MRC-5 and mouse M2-10B4 cells were infected (0.3 or 3 PFU/cell, 16 h) with indicated VACVs, lysed and subjected to western blotting. (**B**) STAT2-HA expressing cells were infected as in (B) for 24 h. Figure 1B is part of a larger experiment which is shown in Figure S6. (**C**) Cells of indicated genotype were infected (5 PFU/cell, 15 h) with wt-VACV or M27-Flag-VACV, lysed and analyzed by western blotting. Please note cross reactivity of the STAT3 antibody for STAT1.

pM27 operates independent of other viral proteins since pM27-Flag expression from a recombinant vaccinia virus (VACV) vector (M27-Flag-VACV), but not wt-VACV or a control VACV, dose-dependently reduced the cellular STAT2 amount in mouse M2-10B4 cells and also in human HeLa (data not shown) and human MRC-5 cells (Figure 1A) indicating that co-factors of pM27 are evolutionary conserved (see below).

To elucidate the molecular mechanism of pM27, we first constructed an expression construct encompassing the coding sequence of the *STAT2* gene devoid of the complete 3′-UTR driven by the constitutive active HCMV major IE promoter. Next, a stably transfected cell line ectopically expressing a C-terminal HA-epitope tagged version of STAT2 complementing STAT2-deficient mouse fibroblasts [15] was generated, designated STAT2-HA, which was permissive for MCMV. The transfectant produced HA-tagged STAT2 at high levels which could be detected either by STAT2- or HA-specific antibodies and became tyr-phosphorylated upon type I IFN treatment, followed by translocation into the nucleus, formation of ISGF3 complexes and induction of IRF-1 expression (Figure S4), indicating a preserved responsiveness and signalling function of STAT2-HA. M27-HA-MCMV, but not ΔM27-MCMV, reduced the amount of STAT2 in STAT2-HA cells (Figure S5), indicating that neither the intrinsic STAT2 promoter nor the 3′-UTR are required for the observed reduction, in accordance with a post-transcriptional mechanism of STAT2 depletion. To confirm the reduction of STAT2-HA, STAT2-HA cells were infected with M27-Flag-VACV, resulting in a loss of STAT2-HA in a time- (Figure S6) and dose-dependent manner (Figure 1B), reproducing the data received with endogenous STAT2. Immunofluorescence staining revealed a decrease of STAT2 amounts upon transfection of *M27* expression plasmids (Figure S7), formally ruling out an intracellular sequestration of STAT2 in detergent resistant compartments.

### pM27 recognizes unphosphorylated and bona fide monomeric STAT2

VACV encodes a multitude of IFN antagonists [16] but does not reduce STAT2 amounts (Figure 1B) while interfering with STAT2 phosphorylation and activation [17]. The ability of VACV-expressed pM27-Flag to affect STAT2 thus suggested that STAT2 is recognized by pM27 in its unphosphorylated and *bona fide* monomeric state. To test this hypothesis further, STAT1-, STAT2-, STAT3- and IFNAR1-deficient cells were infected with M27-Flag-VACV and the relative efficiency of pM27 to reduce the amounts of STAT2 was analyzed. M27-Flag-VACV, but not wt-VACV, induced the reduction of STAT2 in all cells (Figure 1C), indicating that pM27 can recognize non-phosphorylated STAT2 molecules, independent of their incorporation into ISGF3 complexes or previously described STAT3∶STAT2 heterodimers [18].

### MCMV decreases cellular STAT2 amounts by reducing its protein half-life

**Figure 2:**
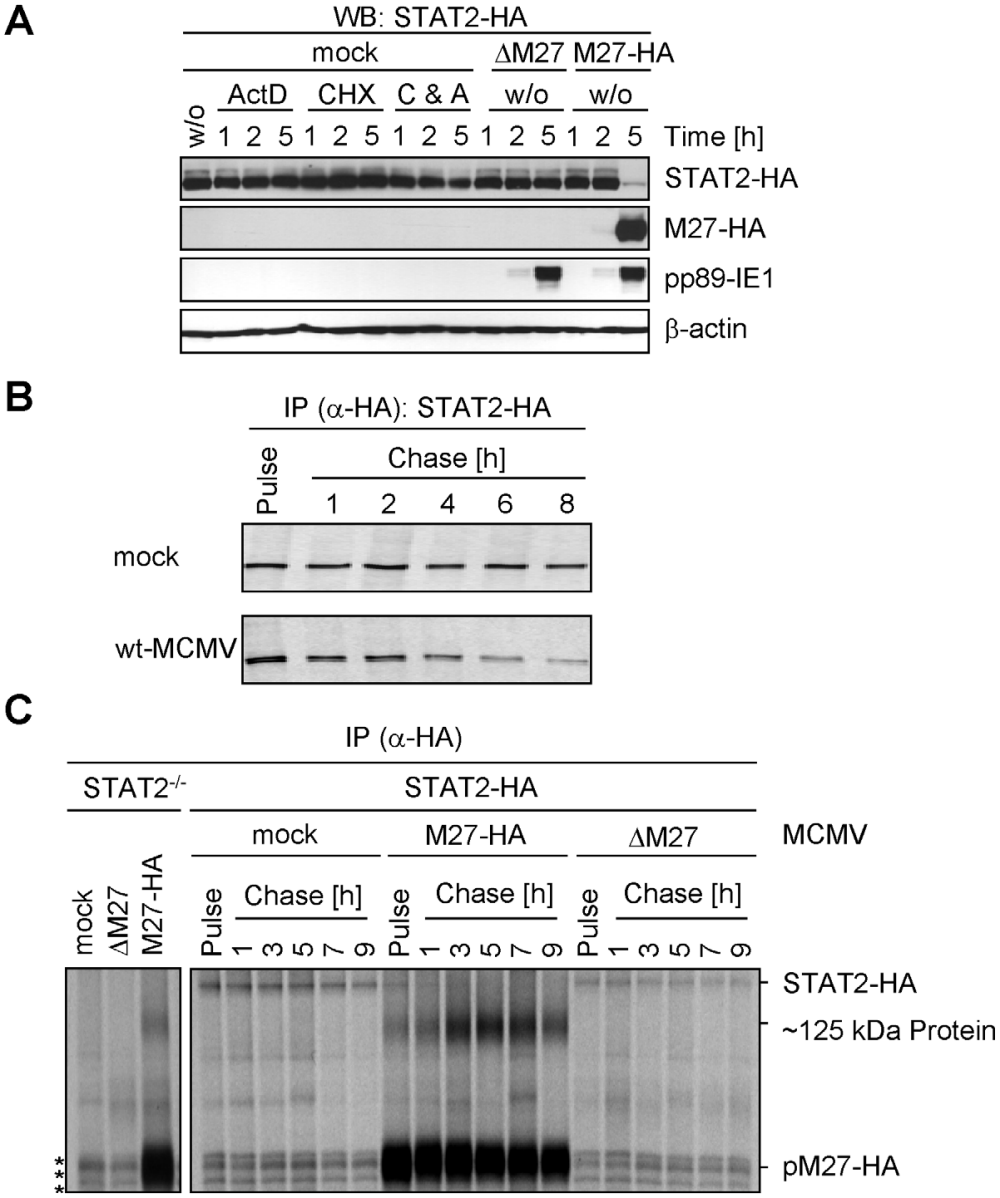
pM27 affects STAT2 protein levels. (**A**) STAT2-HA cells were infected with indicated MCMV mutants (30 PFU/cell). Cells were incubated with or without cycloheximide (100 µg/ml) and actinomycin D (5 µg/ml). After 1, 2 or 5 h cells were analyzed by western blotting. The inhibitors precluded expression of pp89-IE1 and pM27-HA indicating tight inhibition. (**B**) ^35^S-L-Cys/L-Met labelled STAT2-HA cells were infected (5 PFU/cell, 20 h). Cells were metabolically labelled (30 min) and subsequently chased for the indicated time. STAT2-HA was precipitated using an HA-specific antibody. Proteins were separated by 8% SDS-PAGE and visualized by autoradiography. (**C**) As in (B), but cells were infected (20 PFU/cell) for 90 min prior to the indicated pulse-chase regime. Asterisks indicate non-specifically precipitated proteins.

To assess if pM27 affects the pre-existing STAT2 protein pool, STAT2 amounts were compared in presence and absence of pM27 upon administration of the protein synthesis inhibitor cycloheximide (CHX) and the transcription inhibitor actinomycin D (ActD). The pM27-dependent STAT2 reduction preceded the reduction upon blockade of *de novo* protein biosynthesis (Figure 2A). 5 h post MCMV infection STAT2 was hardly detectable whereas combined treatment with CHX and ActD did not significantly affect STAT2 - a finding which is consistent with the previously described long half-life of STAT2 [19]. Next, pulse-chase experiments were performed to compare the STAT2-HA half-life in mock-infected and M27-HA-MCMV-infected cells. Cells were labelled with ^35^S-L-Met/L-Cys and chased for the indicated time (Figure 2B) before the cells were lysed and STAT2-HA protein was precipitated. Upon infection with *M27*-expressing MCMV the half-life of STAT2-HA was strongly reduced when compared to mock-infected cells, which was not observed upon infection with ΔM27-MCMV either (Figure 2C). pM27 protein longevity lasted more than 9 h (Figure 2C). Altogether, the results demonstrated that STAT2 protein stability becomes strongly down-regulated by pM27. Interestingly, an additional long-lived ∼125 kDa protein emerged which was co-precipitated with pM27-HA irrespectively of STAT2 presence (Figure 2C).

### pM27 induces STAT2 ubiquitination and degradation along the ubiquitin proteasome pathway

**Figure 3:**
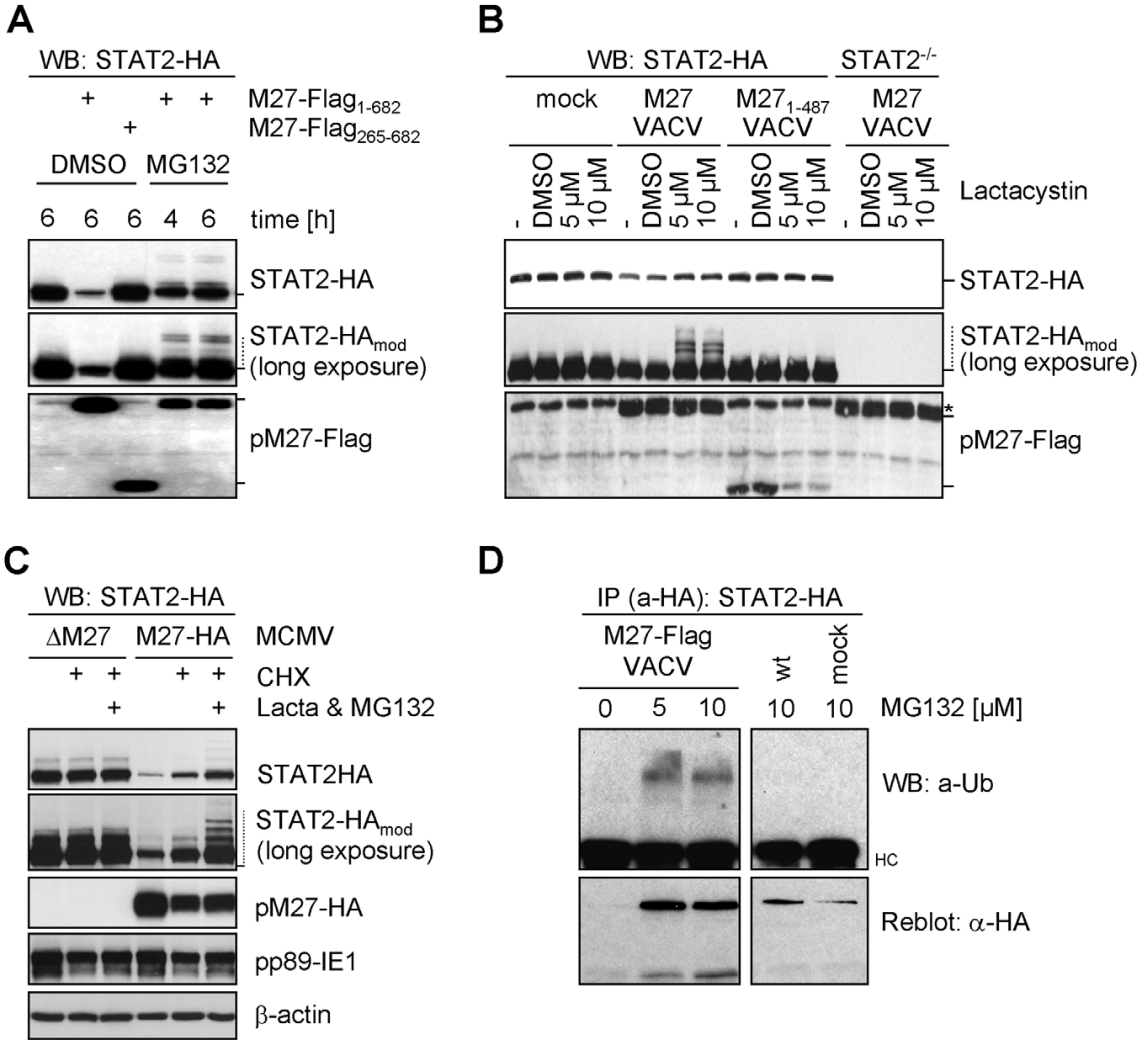
pM27 facilitates the proteasome to degrade STAT2. (**A**) STAT2-HA cells were infected with VACV (3 PFU/cell, 8 h) expressing full-length M27-Flag or a non-functional (M27-Flag_265–682_) truncation mutant and treated for 4 or 6 h with 10 µM MG132 or solvent DMSO and analyzed by western blotting. (**B**) STAT2-HA and STAT2^−/−^ cells were infected (3 PFU/cell, 4 h) with VACV expressing pM27-Flag or a non-functional mutant (M27-Flag_1–487_), treated (4 h) with 5 or 10 µM lactacystin or DMSO and analyzed by western blotting. * indicates an unspecifically detected protein running close to pM27-Flag. (**C**) STAT2-HA cells were infected (20 PFU/cell, 2 h) with ΔM27-MCMV or M27-HA-MCMV and incubated (3 h) with CHX (50 µg/ml) alone or a combined treatment of CHX, lactacystin (10 µM) and MG132 (20 µM). (**D**) STAT2-HA cells were infected (10 PFU/cell) with M27-Flag-VACV or wt-VACV. 4 h post infection MG132 (10 µM) was added. 24 h post infection (M27-Flag-VACV) or 20 h post infection (wt-VACV) cells were lysed and STAT2-HA was precipitated by HA-specific antibody. Precipitated proteins were separated by SDS-PAGE and blotted. The membrane was probed with a Ub-specific and reprobed with an HA-specific antibody.

To investigate whether pM27 uses the Ub proteasome pathway, cells were treated with MG132, an inhibitor of the proteasome. STAT2-HA levels became largely restored and high-molecular weight forms of STAT2 accumulated in the presence of pM27 (Figure 3A). Exploiting the intrinsic *host-shut-off* mechanism of VACV, thereby blocking STAT2 neo-synthesis, we quantified STAT2-HA amounts upon infection with wt-VACV in comparison to M27-Flag-VACV in presence and absence of MG132. The STAT2-HA half-life was drastically reduced by pM27 but could be largely restored upon administration of MG132 (Figure S8). To confirm this phenotype for endogenous STAT2, NIH3T3 cells were infected with M27-Flag-VACV and treated with MG132. As shown in Figure S9, higher molecular weight forms of STAT2 could be detected by a STAT2-specific antibody. When the cells were infected with pM27-encoding VACV only for a short period precluding complete STAT2 degradation, a modification of STAT2-HA was observed in the presence of pM27-Flag and lactacystin, an inhibitor of the proteasome (Figure 3B). The modification was not seen upon expression of a non-functional truncation mutant, pM27 1–487, in untreated or in DMSO solvent-treated cells. The identity of STAT2 was further confirmed by comparison with STAT2^−/−^ cells (Figure 3B).

It has been demonstrated that viral gene expression and genome replication of both CMV and VACV are blocked by inhibitors of the proteasome [20], [21]. To exclude that STAT2 restoration by proteasome inhibitors occurs indirectly due to reduced pM27-HA expression, CHX was co-administrated with MG132 and lactacystin to terminate protein synthesis. Under this regime pM27 amounts remain unchanged upon proteasome inhibition. Nevertheless, restoration and modification of STAT2 was still evident (Figure 3C), indicating that the proteolytic activity of the proteasome is directly required for pM27-induced STAT2 degradation.

To corroborate that the STAT2-modifying moiety is Ub, STAT2-HA cells were infected with M27-Flag-VACV before treatment with MG132. STAT2-HA was precipitated and analyzed using an Ub-specific antibody. As expected, MG132 treatment stabilized the otherwise degraded STAT2 in the presence of pM27-Flag (Figure 3D). Higher molecular weight forms of STAT2-HA were recognized by an Ub-specific antibody in the presence of pM27 and MG132. In conclusion, these results indicate that pM27 induces STAT2 ubiquitination targeting the protein for proteasomal degradation.

### pM27 binds a cellular 125 kDa protein

**Figure 4:**
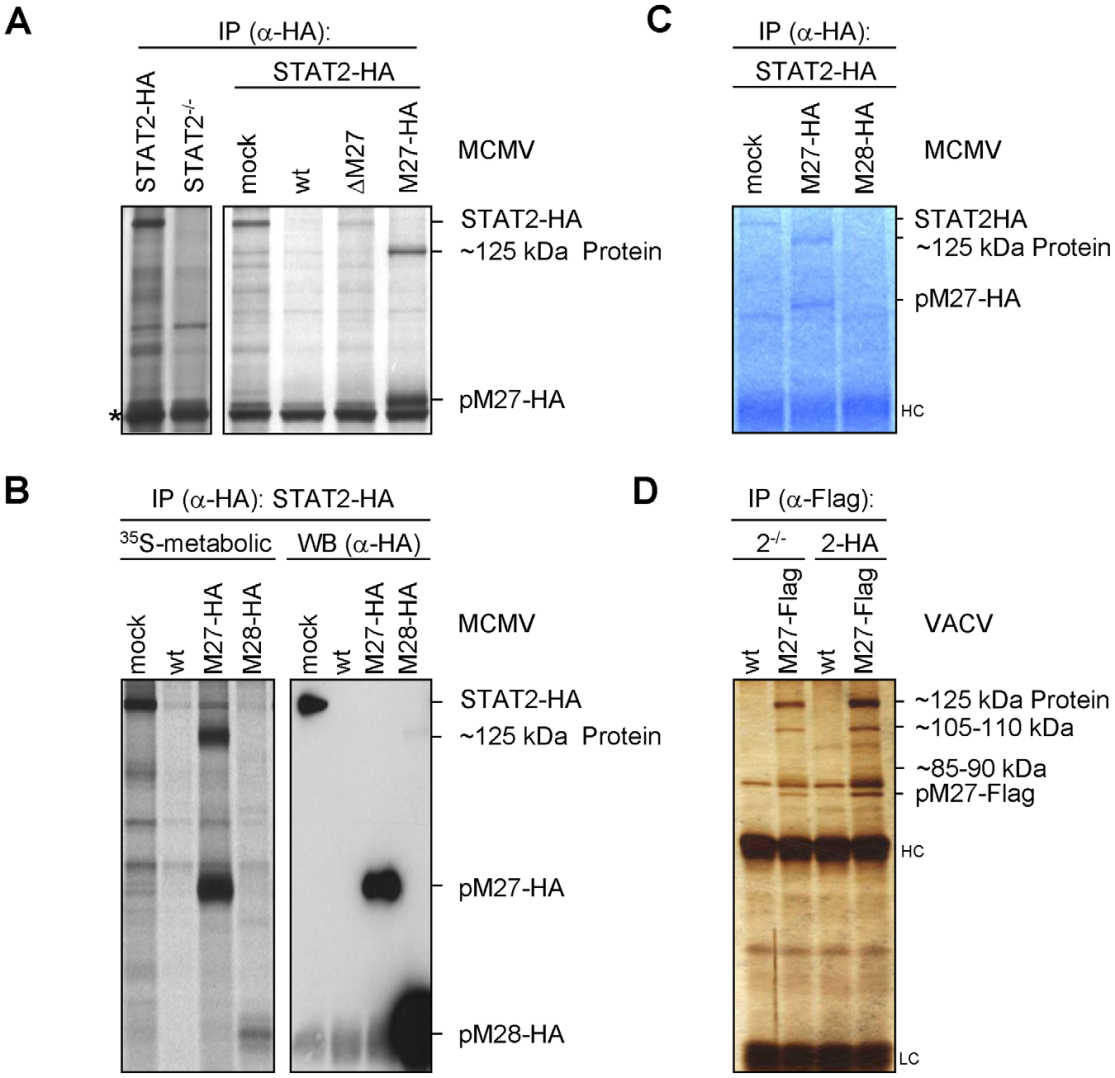
pM27 co-precipitates a ∼125 kDa protein. (**A**) STAT2-HA (2HA) and STAT2-deficient (2^−/−^) cells were infected (5 PFU/cell, 24 h) with wt-MCMV, ΔM27-MCMV, M27-HA-MCMV or left uninfected. Cells were starved (1 h) and then ^35^S-L-Met/L-Cys labelled (150 min). Lysates were subjected to IP using an HA-specific antibody. The asterisk indicates a non-specific protein. (**B**) As in (A), but cells were infected (10 PFU/cell, 48 h) with M27-HA-MCMV, M28-HA-MCMV, wt-MCMV or left uninfected. Lysates were split and analyzed by western blotting in parallel to autoradiography. pM28-HA forms multimers leading to the weak high-molecular weight signal in the rightmost lane. (**C**) As in (A), but cells were infected for 22 h. SDS-PAGE was followed by Coomassie-staining. (**D**) STAT2-HA or STAT2^−/−^ cells were infected (5 PFU/cell, 20 h) with M27-Flag-VACV or wt-VACV, lysed and analyzed by HA-specific IP. Proteins were separated by SDS-PAGE and visualised by silver staining.

As we did not detect sequences or motifs that are characteristical for Ub-ligases within *M27* we surmised that pM27 serves an indirect function to shuttle STAT2 into the Ub-proteasome pathway. To identify potential interaction partners of pM27, a co-immunoprecipitation (IP) strategy was ensued. STAT2-HA cells were infected with M27-HA-MCMV and metabolically labelled. This allowed to follow up the fate of pM27-HA and STAT2-HA simultaneously. By comparing STAT2-HA transfectants with STAT2^−/−^ cells, pM27-HA and STAT2-HA derived co-precipitations could be distinguished. Interestingly, antibodies recognizing pM27-HA specifically co-precipitated a ∼125 kDa protein reproducing the observation made before (compare Figure 4A with Figure 2C). The ∼125 kDa protein was visible after pM27-HA IP but not upon precipitation of pM28-HA (Figure 4B). The co-precipitated protein was also observed in NIH3T3 cells and could be freed by addition of an excess of uncoupled HA-peptides (Figure S10), confirming that it was recovered via an epitope-specific interaction of HA antibodies. Next, split IP samples were simultaneously analyzed by autoradiography upon metabolic ^35^S-Met/Cys-labeling and by anti-HA immunoblotting. The co-precipitated ∼125 kDa protein was visible in the autoradiography but remained undetectable in the immunoblot with HA antibodies (Figure 4B) indicating that it is not derived from pM27-HA.

Upon up-scaling and optimization the co-precipitated protein could be visualized by Coomassie staining of the gels (Figure 4C). The co-precipitating ∼125 kDa protein was also observed upon expression of pM27-Flag by a VACV (Figure 4D) confirming its interaction with pM27. Recovery of the ∼125 kDa protein was achieved in STAT2-HA and in STAT2^−/−^ cells (Figure 4D), ruling out that the protein is STAT2, a degradation product of STAT2 or that STAT2 is required for its interaction with pM27. In summary, these experiments identified the ∼125 kDa protein as a novel cellular co-factor of pM27. Since pM27-Flag co-precipitated further proteins of various sizes (Figure 4D) pM27 was assumed to associate with a cellular multi-protein complex.

### The ∼125 kDa interaction partner of pM27 is DDB1

**Figure 5:**
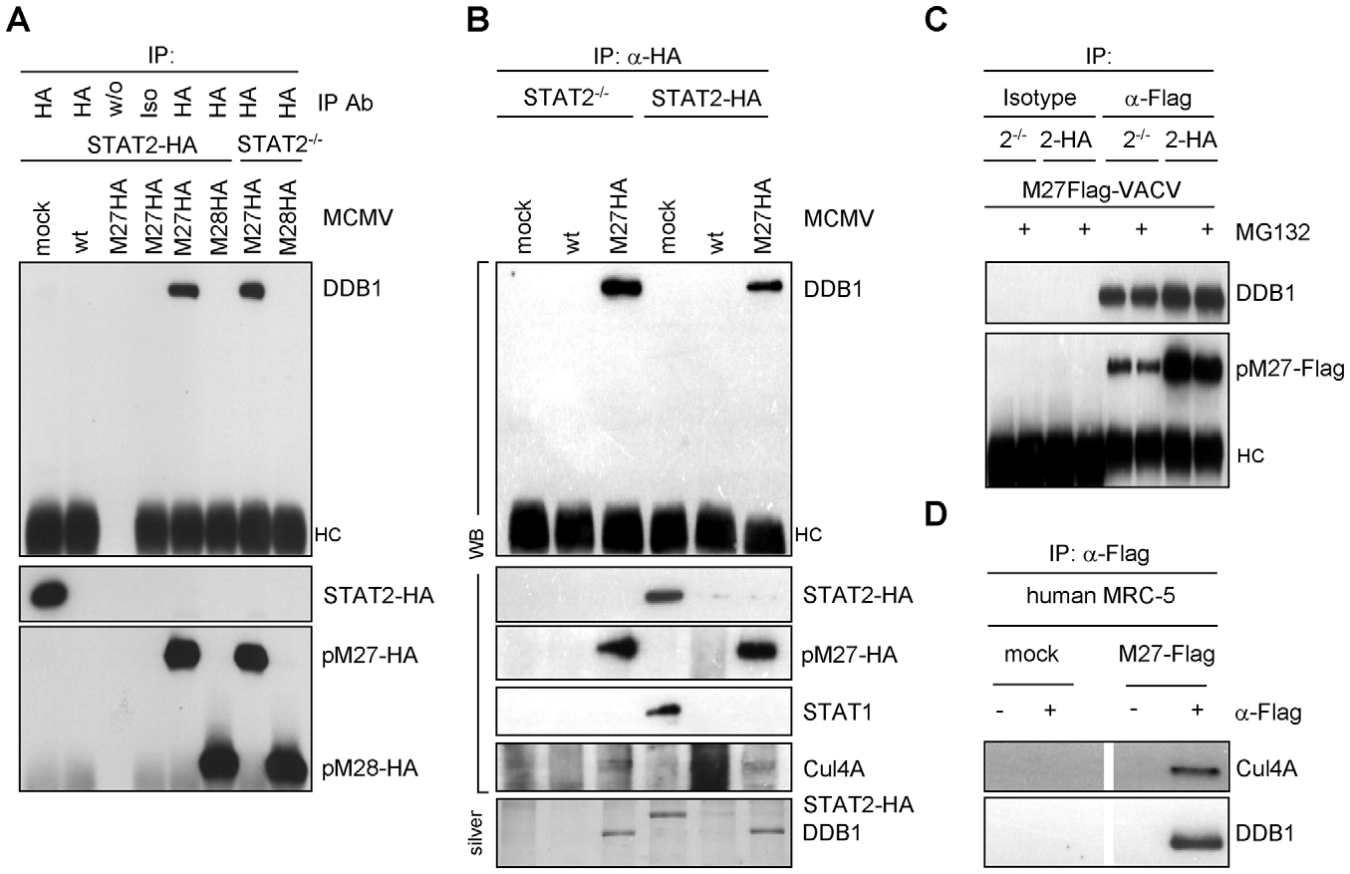
pM27 co-precipitates with DDB1. (**A**) STAT2-HA and STAT2^−/−^ cells were infected (5 PFU/cell, 24 h) with indicated MCMV-mutants (wt-MCMV, M27-HA-MCMV or M28-HA-MCMV), lysed and subjected to IP using HA-specific antibody, an isotype or no antibody. Precipitated proteins were analyzed by western blotting. (**B**) Infection was done as in (A). Cells were lysed and used for HA-specific IP. Precipitated proteins were analyzed by western blotting and by silver staining. (**C**) STAT2-HA and STAT2^−/−^ cells were infected (5 PFU/cell, 18 h) with M27-Flag-VACV, incubated with MG132 (10 µM, for the last 4 h), lysed and subjected to IP using a Flag-specific antibody or an isotype antibody. Precipitated proteins were detected by western blotting. (**D**) MRC-5 cells were infected (5 PFU/cell, 18 h) with M27-Flag-VACV, lysed and analyzed by IP with Flag-specific antibodies. The immune complexes were separated by SDS-PAGE and analyzed by western blotting.

The 125 kDa band was cleaved from a Coomassie-stained gel and analyzed by mass-spectrometry. Five peptides (YLAIAPPIIK, ALYYLQIHPQELR, VTLGTQPTVLR, IVVFQYSDGK and SVLLLAYKPMEGNFEEIAR) were found, all belonging to DDB1, a host 127 kDa protein, concordant with the size of the pM27 co-precipitated material. Two further replications of DDB1-pM27-co-precipitations and subsequent mass-spectrometry analysis reached a peptide coverage rate of 24.8% and 30.2% of the ∼127 kDa full length protein, respectively, unequivocally defining DDB1 as pM27-interacting protein. DDB1 is an adapter protein for the cellular Cul4A-RocA E3-Ub-ligase complex, previously shown to be an interaction partner for paramyxoviral IFN antagonists targeting STAT molecules for proteasomal degradation [22]–[25]. In cells, DDB1 fulfils a function as component of a multimeric ubiquitin-ligase complex involved in nucleotide excision repair and induces ubiquitination of the licensing factor Cdt1 upon UV irradiation [26]. Next, the pM27-DDB1 association was confirmed by immunoblotting with a DDB1-specific antibody upon pM27 immunoprecipitation (Figure 5A). Moreover, STAT2 was not required for the binding of DDB1 by pM27HA (Figure 5A). As expected, DDB1 was not retrieved upon anti-HA IP from cells infected with wt-MCMV lacking the HA epitope fused to the *M27* sequence (Figure 5B). Conversely, DDB1 co-immunoprecipitation was seen with antibodies recognizing pM27-Flag expressed by VACV, irrespective of the presence of STAT2 and in the presence of MG132, confirming that the interaction occurs independently of the epitope tag and the activity of the proteasome (Figure 5C). The retrieval of pM27-HA-DDB1 complexes was pM27-dose-dependent (Figure S11) and resistant to calf intestine phosphatase (CIAP), the phosphatase inhibitor NaF, the detergent CHAPS and tolerated more than 500 mM NaCl and up to 5 mM EDTA (data not shown), reflecting a strong protein-protein interaction.

DDB1 is involved in UV-induced DNA damage responses, and the UV-DDB complex consists of the two separate proteins DDB1-p127 and DDB2-p48 [27]. Hamster cells induce significantly less DNA-binding UV-DDB complexes due to the complete absence of DDB2 [28]. When pM27 was expressed in Chinese hamster ovary (CHO) cells, DDB1 was readily retrieved by co-immunoprecipitation of pM27-HA but not pM28-HA (Figure S12), suggesting that the interaction of the proteins can occur independently of DDB2.

In addition to DDB1 further pM27 co-precipitated proteins were noticed (Figure 4D). Since DDB1 acts as an adapter protein for the Cul4A-RocA complex, we next analysed the co-precipitation of pM27 with the scaffold protein Cul4A which recruits the catalytic RING-finger-containing Ub-ligase RocA. A pM27-Cul4A co-precipitation was weakly visible in mouse cells by immunoprecipitation, presumably due to a poor reactivity of Cul4A antibodies to mouse Cul4A. We therefore expressed pM27 in human cells resulting in a complete STAT2 down-regulation (Figure 1A), reproducing co-precipitation of Cul4A with pM27 and DDB1 (Figure 5D). We concluded that pM27 co-precipitates DDB1 and Cul4A irrespective of the presence of STAT2 or DDB2.

### Mutants of pM27-Flag define the minimal functional and the DDB1 binding domain

**Figure 6:**
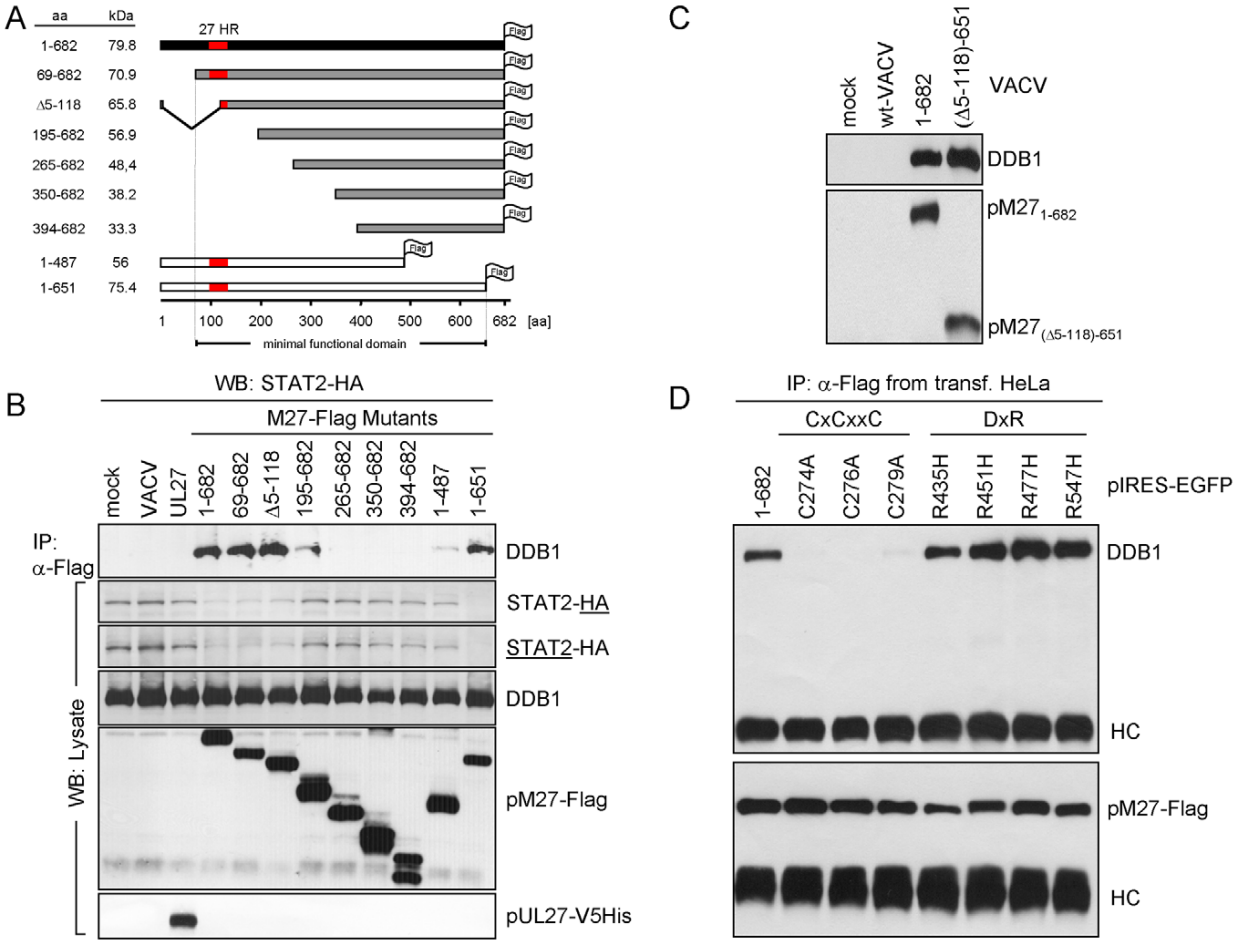
Truncation analysis of pM27-Flag indicates correlation between DDB1 binding and STAT2 degradation. (**A**) The schema depicts the generated *M27* truncation mutants, all constructs are Flag-epitope tagged and expressed by recombinant VACVs. ‘27HR’ indicates a conserved domain shared between homologous proteins of related cytomegaloviruses. (**B**) STAT2-HA cells were infected with the indicated pM27-expressing VACV (16 h; 5 PFU/cell). Proteins from cell lysates were immunoprecipitated using a Flag-specific antibody (upper panel). The precipitated proteins were detected by western blotting. A part of the lysate was acetone precipitated and used to analyse the overall protein amounts (lower panel). (**C**) Cells were infected with wt-VACV or a VACV expressing a pM27-Flag protein, lacking aa 5–118 at the N-terminus and 652–682 at the C-terminus. After precipitation with α-Flag antibody, proteins were analyzed by western blotting. (**D**) pIRES-EGFP plasmids expressing the indicated pM27-Flag mutants were transiently transfected into human HeLa cells and subjected to α-Flag immunoprecipitation and subsequent western blotting using DDB1-specific and Flag-specific antibodies.

To define the essential domain for the interaction of pM27 with DDB1 a panel of Flag epitope tagged truncation mutants of pM27 expressing VACVs was constructed. As depicted in Figure 6A only the truncation of the first N-terminal 68 amino acids and the last C-terminal 30 amino acids were fully dispensable for the ability of pM27 to induce STAT2 degradation. All functional pM27-Flag mutants able to induce STAT2 degradation invariably co-precipitated DDB1 (Figure 6B), revealing a correlation between STAT2 degradation and their DDB1 binding capacity. Mutants lacking the N- or C-terminal 195 amino acids showed a reduced but still detectable binding to DDB1 without being able to degrade STAT2 (Figure 6B). From the fact that neither the first 195 N-terminal nor the last 195 amino acids were essential for DDB1 precipitation we conclude that the minimal DDB1-co-precipitating sequence lies within aa_195–487_ of pM27. To corroborate this finding, we constructed a mutant lacking the N- as well as the C-terminus (pM27-Flag-(Δ5-118)-651). This mutant was still capable to co-precipitate DDB1 upon transient transfection (data not shown) and upon expression from a recombinant VACV (Figure 6C).

Notably, within this minimal functional domain DxR motifs and a conserved CxCxxC motif are present (Figure S13): Binding partners of DDB1 have the consensus motif WDxR, or less frequently YDxR [29], [30]. PM27 contains a WD dipeptide and four DxR sequences, one of them forming the sequence YDxR (aa_544_–aa_547_). We therefore decided to mutate these motifs. Based on the well-described abrogation of DDB1-binding due to a single mutation (R273H) in the DxR motif of DDB2, found in individuals with xeroderma pigmentosum group E ([31]), we mutated the arginine (R) to histidine (H). All four mutant proteins were fully functional in terms of DDB1 co-precipitation (Figure 6D) and in terms of STAT2-degradation (exemplarily shown for R435H –Figure S14) indicating functional redundancy of these sites or that pM27 exhibits an unusual DDB1 interaction.

SV-5 protein, a paramyxoviral DDB1-binding protein, contains two zinc binding pockets critically required for DDB binding [32], one of which with the sequence CxCxxC (aa_206–211_) [33]. Remarkably, a CxCxxC motif is also present in pM27 (aa_274–279_) raising the question if pM27 is also a Zn^2+^-binding protein. Intriguingly, the CxCxxC motif is conserved throughout cytomegalovirus evolution in *M27* homologs with the exception of HCMV and CCMV (Figure S13). We therefore mutated individual cysteins to alanine. All three mutant proteins were impaired in their capacity to co-precipitate DDB1 (Figure 6D) upon transient transfection into HeLa cells and upon expression by recombinant VACVs (Figure S14). Consistent with the hypothesis of DDB1 requirement for pM27-mediated STAT2 degradation, the C279A mutant shows a diminished STAT2 degradation potential (Figure S14).

### pM27 but not its homolog pUL27 binds human DDB1

Like MCMV, HCMV induces a down-regulation of STAT2 in infected cells, which is sensitive to inhibitors of the proteasome. This effect occurs independent of pUL27, the HCMV homolog of pM27 [34]. Consistently, pUL27 expression by VACV neither degraded STAT2-HA nor was sufficient to co-precipitate DDB1 (Figure 6B and Figure S15). In contrast, pM27 readily co-precipitated DDB1 in human cells (Figure 5D), consistent with the high degree of sequence conservation of DDB1 and the functional competence of pM27 in human cells. From this comparative analysis between HCMV and MCMV we conclude that despite the phenotypical match of STAT2 degradation via the ubiquitin-proteasome pathway the genetic and molecular basis between both viruses is remarkably different.

### Knock-down of DDB1 partially restores the STAT2 amount

**Figure 7:**
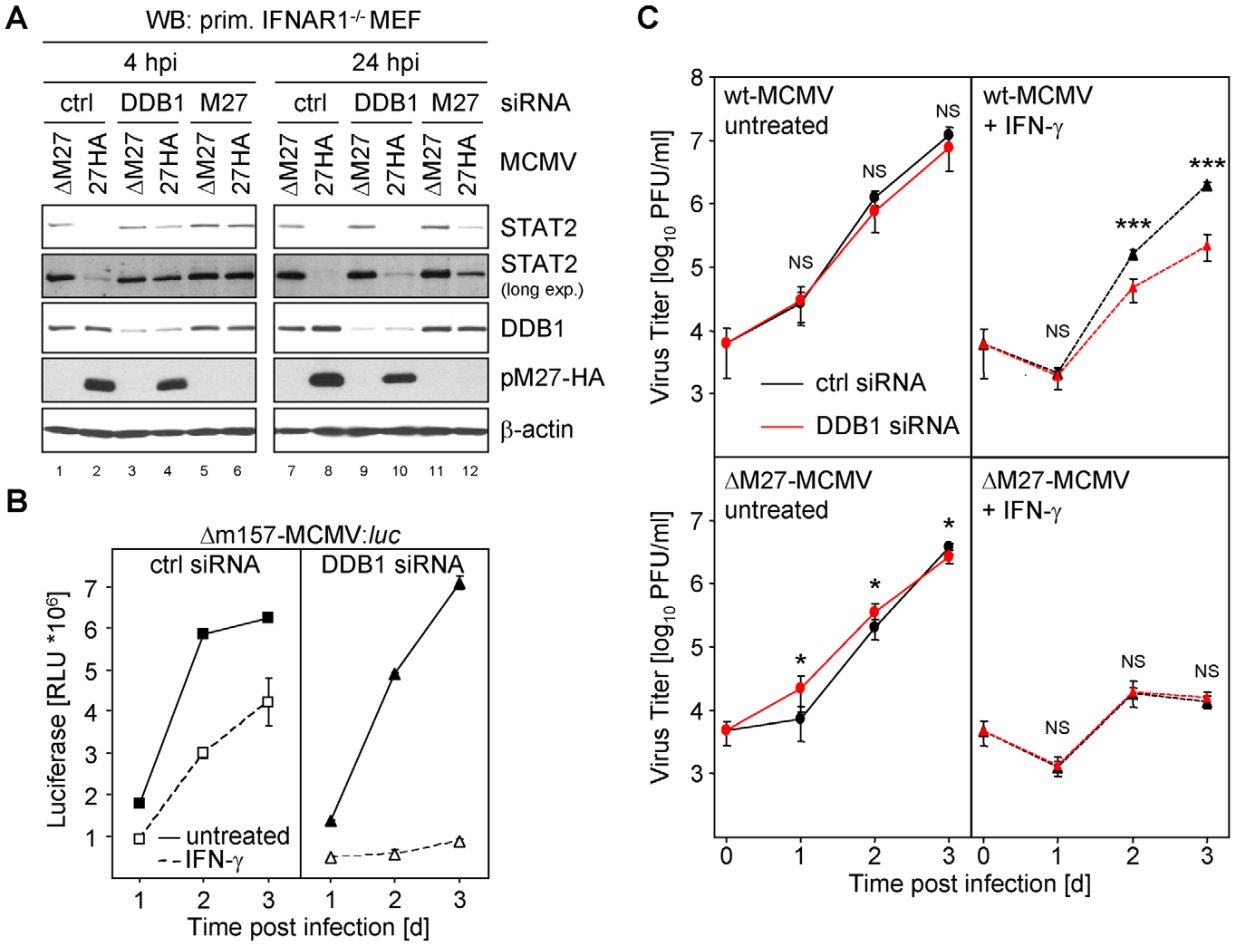
siRNA mediated knock-down of DDB1 restores STAT2 amount and phenocopies *M27*-deficiency. (**A**) Primary IFNAR1-deficient MEF cells (passage 3) were transfected with 200 nM *DDB1*-siRNA, *M27-*siRNA and an irrelevant siRNA, respectively. 24 h later cells were infected for 4 or 24 h with 10 PFU/cell M27-HA-MCMV or ΔM27-MCMV. Cells were lysed and subjected to western blotting. (**B**) Primary MEFs were transfected as in (B), infected (0.1 PFU/cell, 48 h) with Δm157-MCMV:*luc* and luciferase activity was measured. (**C**) As in (C) but cells were infected with wt-MCMV or ΔM27-MCMV and infectious virus progeny was determined by plaque titration.

Recently, a floxed *DDB1* allele has been cloned and recombined into the *DDB1* gene locus in mice. Global Cre-mediated *DDB1* excision results in embryonic lethality [35]. Additionally, conditional DDB1 gene knock-down causes a severe growth defect and apoptosis in the chicken DT40 B cell line [36]. This approach prompted us to carefully ablate DDB1 synthesis by siRNA to analyze the functional relevance of DDB1 for the pM27-dependent down-regulation of STAT2. Transfection of DDB1-specific siRNAs induced a continuous reduction of DDB1 protein amounts (Figure S16). To exclude that siRNA transfection influences the levels of STAT2 due to type I IFN induction, we performed the experiment in IFNAR1-deficient fibroblasts. As expected, infection with M27-HA-MCMV, but not ΔM27-MCMV, induced STAT2 degradation in cells treated with control siRNA. Conversely, siRNA-mediated knock-down of pM27 restored STAT2 (Figure 7A, lanes 6 & 12). Likewise, DDB1 ablation fully restored STAT2 amounts 4 h post infection (lane 4) and partially after 24 h (lane 10). Consistent results were obtained upon pM27 expression from VACV (data not shown). These findings establish that DDB1 is a prerequisite to execute effective STAT2 proteolysis by pM27.

### siRNA-mediated knock-down of DDB1 phenocopies M27-deficiency

*M27*-positive MCMVs antagonize the induction of IFN-γ-stimulated, STAT2-containing, ISRE-DNA-binding complexes (Figure S17). Consistently, replication of the ΔM27-MCMV mutant is characterized by its enormous susceptibility towards IFN-γ *in vitro* and *in vivo* [11]. To test whether DDB1 is relevant for this effect, we transfected MEF with DDB1-specific- (or control-) siRNAs 48 h prior to infection before the cells were incubated with IFN-γ24 h prior to infection. The MCMV infection was performed with a luciferase expressing mutant, Δm157-MCMV:*luciferase* in which the coding sequence of *m157* has been replaced by the *luciferase* gene, and cells were harvested 1, 2 and 3 days post infection. Luciferase activity paralleled the kinetics of MCMV replication. Accordingly, luciferase activity was inhibited upon IFN-γ pretreatment of MEF (Figure 7B, left panel). While DDB1 knock-down precluded viral luciferase expression by the *M27*-positive Δm157-MCMV:*luciferase* mutant in IFN-γ preincubated cells, luciferase production was unaffected in cells which were not IFN-treated (Figure 7B). The DDB1 knock-down and reduced viral gene expression was confirmed by western blot analysis of cell lysates using DDB1- and pp89-IE1-specific antibodies (data not shown). The experiment was repeated with wt-MCMV and the progeny virus yield was quantified by standard plaque titration. IFN-γ pre-treatment of cells, which had been treated with DDB1-specific siRNA, strongly impaired MCMV growth (Figure 7C). In clear contrast, the replication of ΔM27-MCMV was highly susceptible to IFN-γ and was not further impaired by ablation of DDB1 by siRNA (Figure 7C, lower panel). Altogether, these data indicate that DDB1 by itself is not required for MCMV replication. However, the virus requires DDB1 to overcome the STAT2-dependent antiviral capacity of IFN-γvia pM27. The phenocopy of host DDB1 depletion and viral *M27*-deletion provides complementary evidence for a model in which DDB1 is indispensable for pM27 subversion of the antiviral IFN-γ response.

## Discussion

**Figure 8:**
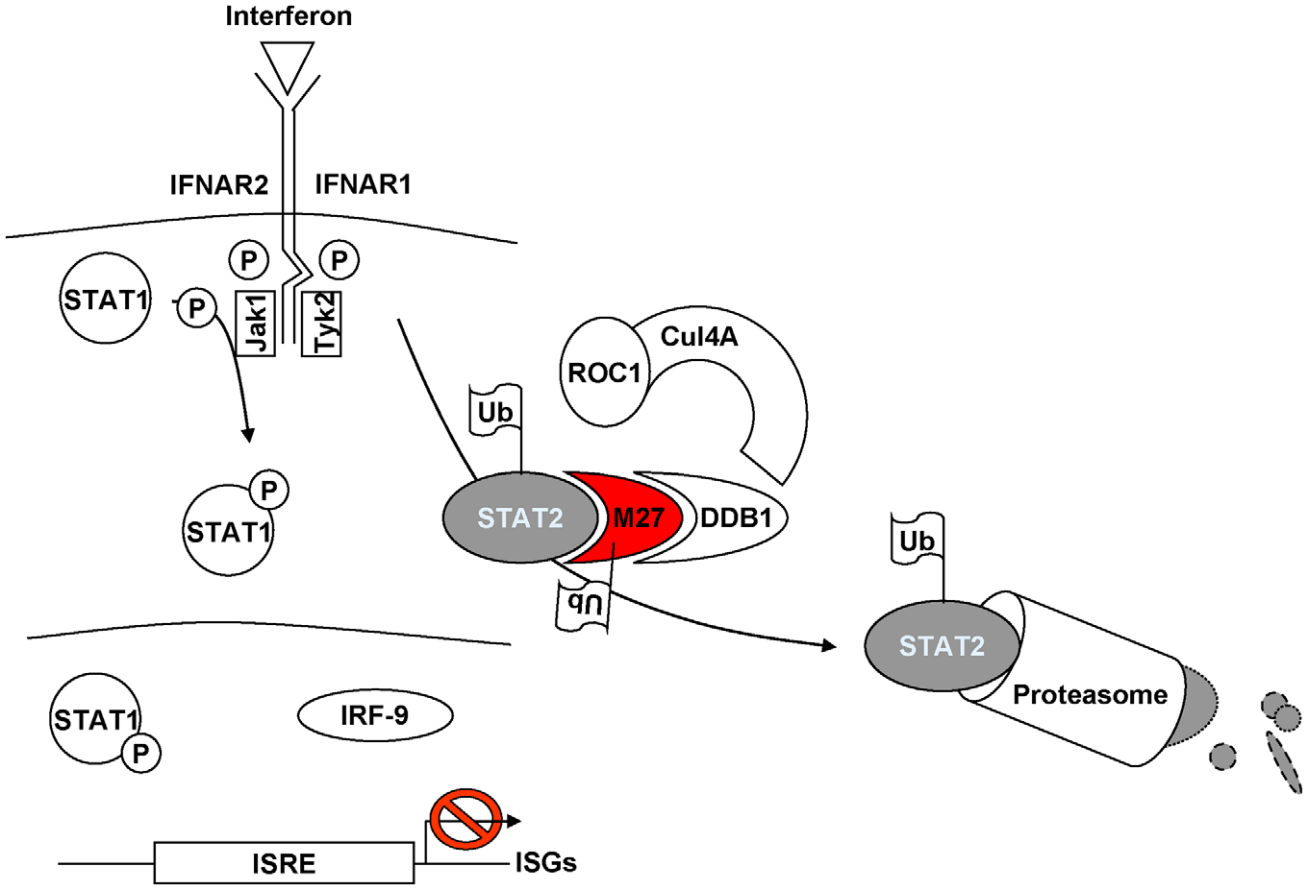
Current model of pM27 function. pM27 bridges STAT2 to DDB1-containing ubiquitin-ligase complexes thereby inducing poly-ubiquitination and subsequent proteasomal degradation of STAT2 to antagonize induction of interferon stimulated genes.

In this study we have elucidated the mechanism of the cytomegaloviral IFN antagonist pM27. This viral protein is essential and sufficient for the MCMV-encoded inhibition of the IFN signalling cascade by binding and degrading STAT2, a transcription factor which becomes activated in the type I and type II IFN receptor signal transduction. Replication of ΔM27-MCMV is highly attenuated in IFN-treated cells and in infected mice [11], [12] indicating that STAT2 initiates an efficient antiviral effector program unless its degradation is accomplished. pM27 is demonstrated to degrade STAT2 via the ubiquitin proteasome pathway by binding to DDB1 (Figure 8: Model of pM27 function). This host factor is per se not required for MCMV replication, but becomes conditional essential in the presence of IFN-γ. From these findings we infer a crucial role of DDB1 for MCMV replication *in vivo*.

### Implications of DDB1 as a cofactor for viral replication

Recently, genome-wide siRNA-based large-scale screening approaches have been conducted to uncover host factors required for replication of certain viruses including HIV and influenza [37]–[39], representing new potential targets for antiviral therapy. Despite the fact that relevant factors were successfully identified, these attempts suffer from two common shortcomings. First, the implicit counter-selection against siRNAs which are detrimental for cell survival, i.e. a screening bias against ‘essential’ host proteins. It is tempting to speculate that those ‘essential’ proteins are exactly the host factors many viruses favour as interaction partners due to their evolutionary conservation and the inability of the host to mutate or delete the responsible genes. We feel that our results exemplify the fundamental need to pursue ‘top-down’ approaches to refine biological observation (e.g. the growth attenuation of ΔM27-MCMV upon conditioning with IFN-γ) allowing the characterization of underlying molecular mechanisms and finally the identification of (conditional) essential host factors. We were surprised to see that viral infection (presumably due to control over cell cycle progression and apoptosis), increased the ability of cells to resist knock-down of DDB1, raising the apparent question whether it might be reasonable to conduct above mentioned siRNA screens without any previous negative pre-selection. Second, our study documents that distinct host factors are not constitutively essential but become essential under certain conditions defined by the host cell environment, e.g. the IFN-induced antiviral state. It is well possible that only the simulation of conditions which are closer to infected and inflamed organs leads to additional induced essential host factors important for viral replication because they escaped the screening performed under standard cell culture conditions.

### pM27 interaction with STAT2

pM27 has adopted a remarkable substrate specificity to capture its cellular target, monomeric STAT2 [11]. Several findings are fully in accord with the notion that the down-regulation of STAT2 is achieved via the ubiquitin-proteasome pathway: i) pM27 affected the half-life of STAT2, ii) STAT2 reduction was sensitive to proteasome inhibitors, iii) in the presence of proteasome inhibitors pM27 generated higher molecular weight forms of STAT2, and iv) the modification of STAT2 was shown to be conjugated Ub. Given the long protein half-life of STAT2 catalyzing its proteolytic destruction represents a direct and immediate mechanism to shut off its antiviral function. The recognition and binding of STAT2 requires a large and central domain of the pM27 protein as revealed by probing of a set of truncation mutants.

### pM27 interaction with DDB1

Co-IP studies revealed the prominent binding quality of pM27 to a second host protein which was identified to be DDB1. Forming an adaptor protein of the Cul4A-RocA Ub-ligase complex, the linkage of pM27 with DDB1 generated the hypothesis that pM27 delivers STAT2 to proteasomal destruction via this factor. Two findings support the notion that DDB1 is indeed required for the loss of STAT2 in MCMV-infected cells: i) truncation mutants of pM27 induced the break-down of STAT2 only when their binding to DDB1 was fully intact; ii) siRNA-mediated knock-down of DDB-1 protected STAT2 from degradation.

Binding partners of DDB1 have the consensus motifs WDxR, or less frequently YDxR [29], [30]. pM27 contains a WD dipeptide and four DxR sequences, one of them forming the sequence YDxR (aa_544_–aa_547_). Nevertheless, single R>H mutations of the DxR motifs did not impair DDB1 co-precipitation. This might either indicate functional redundancy or that pM27 exhibits an unconventional DDB1-binding mode.

Based on experimental data obtained in the fission yeast (*Schizosaccharomyces pombe*), a so called DDB1-box has been defined to be present in DDB1 binding partners like WDR21 and comprising a RQLG-like motif surrounded by hydrophobic amino acids in positions −7 to −3 and +7 or +9 [40]. PM27 bears two non-identical motifs, which resemble this DDB-box within aa_232–256_ and aa_358–377_, overlapping with the domain that is required for degradation of STAT2. Future analysis will define further essential amino acids which are critical for DDB1-pM27-complex formation and might delineate the molecular requirements for recruitment and exploitation of DDB1-Cul4A-RocA complexes.

The finding that the CxCxxC motif is important for DDB1 co-precipitation suggests that pM27 harbours a coordinative Zn^2+^ binding pocket. Interestingly, this domain is conserved in different cytomegaloviruses (Figure S13), raising the apparent question whether the basic function of the pM27 homologs, proteasomal degradation, might also be conserved.

### pM27 – not just a paramyxoviral analogue device

At the first glimpse pM27 seems to imitate paramyxoviral SV-5 V-proteins which recruit DDB1 and induce proteasomal degradation of STAT proteins. Neither pM27 nor SV-5 V-protein contain a fully conserved WDxR motif. Besides the CxCxxC motif, pM27 and the SV-5 V-protein are considerably different with regard to structure, function and substrate recognition and they do not share homologous amino acid stretches. V-proteins discriminate between human and mouse STATs and require the presence of both STAT1 and STAT2 to induce the degradation of the other [41], [42], whereas pM27 induces the selective degradation of human and mouse STAT2 as a monomer. Several biological observations further imply differences in their molecular functions. pM27 does not affect the induction of type I IFN [14] contrasting with V-proteins [43], [44]. Stable expression of pM27 was not possible (M. Trilling, unpublished observation) but was readily achieved for SV-5 V-protein [45], suggesting a different mode of interaction with DDB1 which is essential for cell survival [36]. MCMV can arrest the cell cycle of infected fibroblasts both in G_1_ and in G_2_ [46]. Since DDB1 is required especially for proliferating cells [35], an attractive hypothesis would be that MCMV can afford a blockade of DDB1 functions due to its ability to arrest the cell cycle prior to the DDB1-sensitive checkpoint. In line with this hypothesis, DDB1 knock-down did not abrogate MCMV replication in MEF by itself, but became strongly antiviral if cells were pretreated with IFN-γ. Given that DDB1 is expressed ubiquitously in all mouse tissues [35] the conditional exploitation of DDB1 by a proviral protein like pM27 appears to be a perfect strategy which combines the need for a broad cell tropism to establish ‘replication factories’ in a large variety of tissues with the defence against the permanent encounter of omnipresent IFN-γ which is produced in response to the herpesviral life style bringing sustained immune exposure.

## Material and Methods

### Cells and cytokines

MRC-5 (ATCC CCL-171), M2-10B4 (ATCC CRL-1972), immortal STAT2^−/−^- [15] and STAT1^−/−^ mouse fibroblasts [47], crisis immortalized IFNAR1-deficient (generated from primary IFNAR1-deficient MEF [11]) and primary MEF (prepared as described [48]) were grown in Dulbecco's modified eagle medium (D-MEM) with 10% foetal bovine serum, streptomycin, penicillin and 2 mM glutamine. NIH3T3 cells were grown in 10% newborn calf serum. STAT2-HA cells were generated from STAT2^−/−^ cells [15]. STAT2-HA [11] was subcloned into a pcDNA3.1 (Invitrogen)-derived pcDNA3.1-zeocin expression vector. Cell lines were selected under 200 µg/ml zeocin (Invitrogen). IFN-γ (#12500-1) was purchased from PBL Biomedical Laboratories, New Jersey, USA. Inhibitors of the proteasome (MG132 and lactacystin) were purchased from Boston Biochemicals, USA.

### Viruses and plasmids

swt-like MCMV MW97.01, ΔM27-MCMV, M27-HA-MCMV, M27-Flag-VACV and STAT2-HA-VACV have been described [11]. M28-HA-MCMV was generated by amplifying a frt-site flanked *kana^r^*-cassette using primers containing M28-homologous sequences prolonged by an HA-epitope encoding sequence (underlined): AZ-M28-HA1: TGCGGGCTCCGTCCGGGATAGCCGAGACCTGCGTGCCCACGCTCGGGTACCCATACGATGTTCCAGATTACGCGTGACCAGTGAATTCGAGCTCGGTAC and AZ-M28-2: AGGCGAGGCGAAACTGGCGGGATAACTGCAAGAGAGGGGAAAAGCGGTCGATCCCAGCCGGACCATGATTACGCCAAGCTCC using pFRT1 as template. The PCR fragment was introduced into the MCMV-BAC by homologous recombination in *E.coli*. The *kana^r^*-cassette was excised from the BAC by FLP-mediated recombination. *m157* was deleted accordingly by zeocin selection after replacement of the *m157* coding sequence against a *zeo^r^*-cassette by homologous recombination between the MCMV-BAC and a PCR amplificate generated with the primers: AZ-m157-1-CAGGAGAATCTGAACCCCGATATTTGAGAAAGTGTACCCC GATATTCAGTACCTCTTGAC CCAGTGAATTCGAGCTCGGTAC and AZ-m157-2-AGATCGTGACCATTATCACCAAGATAGTTCCCACCATAATTCCCATCGTCACTAGAGTCGGACCATGATTACGCCAAGCTCC and pFRT-Zeo as template. Afterwards *zeo^r^* was replaced by the *luciferase* gene (derived from pTA-luc [Clontech]) by homologous recombination between the Δm157-MCMV:*zeo^r^* BAC and a vector, harbouring a *luciferase* gene flanked by 800 nts of the MCMV genome, surrounding the *m157* coding sequence. BAC-derived MCMV mutants were reconstituted in primary MEFs und correct mutagenesis was confirmed by restriction fragment pattern analysis and PCR (data not shown).

Truncation mutagenesis of pM27-Flag-VACV was performed based on the described VACV expression plasmid p7.5k131-M27-Flag. The C-terminal sequence of the M27 ORF was amplified with the Az-M27-m1_forw: 5′-CAGAAGATCGGCACGAAGTACC-3′ primer with either the MF-M27-m2_rev: 5′-CGCGCGACTAGTCTCGTTGTCGTCGTCCTCGTAG-3′ or - MF-M27-m4_rev: 5′-CGCGCGACTAGTGGAGCCCGACGAATCCTTGTC-3′. Amplificates were cleaved by *BamH*I and *Spe*I (underlined, primer intrinsic site) and cloned into p7.5k-M27Fl/SphI vector between an N-terminal fragment of M27 and an in-frame C-terminal Flag-epitope.

For N-terminal truncations, M27-intrinsic restriction sites (*Apa*I, *Sac*II, *Pvu*I, *Nco*I, *Bam*HI and *Msc*I) were used together with a vector intrinsic *Bgl*II site. After re-ligation the next ATG in frame served as start codon. The pM27-Flag-(Δ5-118)-651 mutant was constructed by replacing the C-terminal part of the Δ5-118 ‘*Sac*II’ mutant with the truncated C-terminal sequence using an internal *Bam*HI site. VACV mutants were selected with BrdU in tk^−^143 cells.

Site-directed mutagenesis of pM27-Flag was performed using the Quick Change kit (Stratagene) according to the instructions of the manufacturer using the following primers and its respective reverse complementary primers: KL-C274A: 5′-catctacgatcaactcGCGtactgtcgcgagtgtc-3′, KL-C276A: 5′-cgatcaactctgttacGCGcgcgagtgtcggatgc-3′, KL-C279A: 5′-gttactgtcgcgagGCGcggatgcgccgggg-3′, KL-R435H: 5′-gcgacgtcgacgccCACatccgcgcgggagc-3′, KL-R451H: 5′-gtcgcctccgaccccCACcaggacggcatctcg-3′, KL-R477H: 5′-caccttctcggacgagCACcccgacggctacgagg-3′ and KL-R547H: 5′-gaggatgtacgacgagCACccgctggccggcttc-3′. Mutations were confirmed by sequencing.

UV inactivation of viruses (MCMV and VACV) was done by exposing viruses for 25 min to UV light (254 nm) from a light source 10 cm afar.

### Western blotting

Cells were lysed in RIPA^+^-buffer (50 mM Tris-HCl, 150 mM NaCl, 1% [vol/vol] IGEPAL, 1% Na-Deoxycholate [vol/vol], 0.1% [weight/vol] SDS, 1 mM DTT, 0.2 mM phenylmethylsulfonyl fluoride (PMSF), 1 µg/ml leupeptin, 1 µg/ml pepstatin, 50 mM NaF, 0.1 mM Na-vanadate with Complete protease inhibitors (Roche) pH 7.5). Samples were normalized according to Bradford protein staining and equal amounts were subjected to denaturing SDS-PAGE. Gels were blotted on nitrocellulose membranes (Schleicher and Schuell) and probed with indicated antibodies. The same membrane was used and consecutively stripped with reblot solution (Calbiochem). The following commercially available antibodies were used: α-β-actin,α-Flag M-2 and α-HA from Sigma-Aldrich; α-IRF-9, α-STAT1, α-mSTAT2, α-STAT3 from Santa Cruz; α-Cul4A (Acris), α-DDB1 (Bethyl), α-pp89-IE1 (Croma101, kindly provided by Stipan Jonjić, Rijeka, Croatia), α-hSTAT2 (Upstate) and α-Ub (Dako).

### Immunoprecipitation

Immunopreciptation was done as described. Briefly, cells were lysed (lysisbuffer: 0.1 mM EDTA; 200 mM NaCl; 10 mM KCl; 10 mM MgCl_2_; 10% [vol/vol] glycerol; 20 mM HEPES [pH 7,4]; 0.5% [vol/vol] IGEPAL; 0.1 mM PMSF; 1 mM DTT; 0.4 mM pepstatin A; 0.1 mM Na-vanadate; Complete protease inhibitor (Roche)). Lysates were spun (30 min at 4°C and 16000 g) and IP antibody was added to the supernatant. Immune complexes were precipitated with Protein-G-Sepharose (Amersham). The pellet was washed by 6–10 consecutive rounds with lysis buffer.

For metabolic labelling and pulse-chase experiments cells were starved (30 min) in L-Met-/L-Cys-free media and subsequently pulsed (90 min) with ∼10 MBq/∼10^6^ cells EasyTag Express ^35^S protein labelling mix (PerkinElmer). After the pulse cells were washed 3 times with chase media (10%-FBS D-MEM supplemented with 1.5 mg/ml L-Met/L-Cys) and chased as indicated. Immune complexes were separated by SDS-PAGE. Gels were either stained by silver- or Coomassie-staining or fixed, dried and visualized by autoradiography.

### siRNA Transfection

2.5–7.5 * 10^4^ primary MEF cells were transfected with siRNA using RNAiMax transfection reagent (Invitrogen) following manufacturers instructions. The siRNAs were purchased from IBA. The following siRNAs were used for the knockdown: DDB1 (5′-[PO_4_] r(AACCUGUUGAUUGCCAAAAACTT)-3′), luc-siRNA (5′-[PO_4_] r(CUUACGCUGAGUACUUCGATT)-3′) and M27 (5′-[PO_4_] r(CAAUAAGCCCUUUAAUCAC)dTdT-3′).

## Supporting Information

Figure S1 M27-HA-MCMV and M28-HA-MCMV, but not ΔM27-MCMV, reduces STAT2 amounts. NIH3T3 cells were infected with wt-MCMV, UV-irradiated MCMV, indicated MCMV mutants (5 PFU/cell, 48 h) or left uninfected. Cell lysates were adjusted and subsequently analyzed by western blotting with specific antibodies as indicated. All proteins were detected on one membrane in iterated rounds of detection.
(TIF)

Figure S2 Quantification of STAT2 amounts in MCMV-infected cells. NIH3T3 cells were infected (10 PFU/cell) with ΔM27-MCMV, wt-MCMV or left uninfected. 4, 8 and 24 h post infection cells were lysed and the endogenous STAT2 amounts were determined by western blotting. Three western blots have been densitometrically quantified. The arithmetic mean ± SD of the relative STAT2 amount compared to mock-infected cells at the same timepoint is shown in a log_10_ scale.
(TIF)

Figure S3 M27 reduces STAT2 even upon induction by IFN-γ (**A**) Schematic overview of the experimental setup. (**B**) Cells were incubated for 48 h with 500 U/ml IFN-γ prior to infection with wt-MCMV or ΔM27-MCMV (10 PFU/cell) for additional 48 h. Subsequently, cells were incubated for 1 h with 250 U/ml IFN-α or IFN-γ before lysis. Lysates were normalized and analyzed by SDS-PAGE and western blotting with the indicated antibodies.
(TIF)

Figure S4 STAT2-HA transfectant express a functional STAT2. (**A**) STAT2-HA becomes tyrosine phosphorylated upon IFN stimulation. BALB/c MEF, STAT2^−/−^ cells and STAT2-HA transfectants thereof were incubated with 50 or 500 U/ml IFN-α for 25 min. Cells were lysed and subjected to western blotting. (**B**) STAT2-HA forms ISGF3 complexes. Cells were stimulated with 25 U/ml IFN-β and native lysates were prepared and analyzed by electromobility shift analysis (EMSA) with a ^32^P-labelled ISRE probe as described before [11]. ISGF3 complexes were super-shifted upon addition of an HA-specific antibody. (**C**) STAT2-HA translocates in the nucleus upon IFN stimulation. STAT2-HA cells were incubated for 1 h with 500 U/ml IFN-α and IFN-γ. Additionally, leptomycin B (LMB) was administered as indicated to block the CRM-dependent protein export. Cells were fixed in 3% (v/v) PFA, permeabilized with 0.2% (v/v) Triton-X-100. STAT2-HA was detected with an HA-specific rabbit antibody and visualized with a Cy3-coupled secondary anti-rabbit antibody. (**D**) IRF-1 can be induced in STAT2-HA cells by IFN-α. STAT2-HA cells were incubated with grading concentrations of IFN-α and induced amounts of IRF-1 were detected by western blotting.
(TIF)

Figure S5 M27-HA-MCMV reduces STAT2-HA amounts whereas ΔM27-MCMV does not. STAT2-HA cells or the corresponding STAT2-deficient parental cells were infected (14 h; 5 PFU/cell) before CHX was added. Cells were directly lysed (Pulse) or at 3, 6 and 12 h post CHX addition and the lysates were analyzed by western blotting using the indicated antibodies.
(TIF)

Figure S6 Loss of STAT2-HA upon infection with M27-Flag-VACV is time- and dose-dependent. A section of this experiment is shown in Figure 1B. STAT2-HA cells or the corresponding STAT2-deficient parental cells were infected with the indicated VACVs. Cells were lysed at indicated timepoints and subjected to western blotting.
(TIF)

Figure S7 Transfection of *M27*-expression plasmids reduces STAT2 amounts on the level of an individual cell. STAT2-HA cells were transfected with a pM27-Flag expression plasmid (pIRES_EGFP_) using Superfect (Qiagen) transfection reagent, 24 h later fixed (3% [v/v] PFA), permabilized (0.2% [v/v] Triton-X-100) and stained with α-HA (rabbit), α-Flag (mouse) and secondary anti-rabbit (Cy3-coupled) or anti-mouse (Cy5-coupled) antibodies. IFM was done as previously described [14]. *M27*-transfected cells are indicated by white arrowheads.
(TIF)

Figure S8 pM27-dependent reduction of the STAT2-HA half-life is MG132 sensitive. STAT2-HA cells were infected with wt-VACV, M27-Flag-VACV or left uninfected. 3 h post infection MG132 (20 µM) was added and cells were lysed at indicated time points. Three experiments were performed and densitometrically quantified. Shown is the arithmetic mean ± SD of the relative STAT2-HA amount compared to untreated wt-VACV infected cells. In the lower panel one representative western blot is shown. High molecular weight forms of STAT2 become apparent in the long exposure.
(TIF)

Figure S9 pM27 induces higher molecular weight forms of endogenous STAT2 upon incubation with MG132. NIH3T3 cells were infected with M27-Flag-VACV or left uninfected. Cells were incubated with 5 or 10 µg/ml MG132. Cells were lysed and lysates were subjected to western blotting with a STAT2-specific antibody.
(TIF)

Figure S10 pM27-HA-DDB1 co-precipitation by HA-specific antibodies occurs epitope specific. NIH3T3 cells were infected with indicated mutants (24 h, 5 PFU/cell) or left uninfected. Cells were lysed and lysates were subjected to an immunoprecipitation with HA-specific antibody. Pellets of sepharose with retrieved immune complexes were incubated with an excess of HA-peptide (200 µg/ml in 10 mM Tris-HCl pH 8.0 for 25 min at 37°C). Supernatants were subjected to 8% SDS-PAGE and silver staining (right panel). Pellets were boiled in sample loading puffer and also analyzed by silver staining (left panel). LC, light chain, HC heavy chain of antibodies.
(TIF)

Figure S11 pM27-DDB1 co-precipitation and STAT2-degradation occur dose-dependently. STAT2-HA cells were infected with grading titers of M27-HA-MCMV. To ensure identical infection dose, ΔM27-MCMV was added to end up with a constant infectious dose of 10 PFU/cell. Cells were lysed and subjected to an anti-HA immunoprecipitation and subsequent western blotting with the indicated antibodies.
(TIF)

Figure S12 pM27 co-precipitates DDB1 in hamster CHO cells lacking DDB2. CHO cells were infected (24 h, 10 PFU/cell) with M27-HA-MCMV or M28-HA-MCMV or left uninfected. Cells were lysed and analyzed by immunoprecipitation with an HA-specific antibody. Immune complexes were analyzed by SDS-PAGE and western blotting with either an HA-specific or a DDB1-specific antibody.
(TIF)

Figure S13 Alignment of CxCxxC motif in cytomegaloviruses. Sequences of indicated cytomegaloviral ‘27’ proteins were inspected for presence of CxCxxC motifs. Shown is an alignment of the 5 known MCMV pM27 sequences (WP15A, C4A, G4A, K181 and Smith) in comparison to the sequences derived from rat CMV (Maastricht), aotine CMV, tupaia CMV (pT27), guinea pig CMV (GpCMV), squirrel CMV, rhesus CMV (rhCMV), chimpanzee CMV (CCMV), human CMV (HCMV pUL27) and the SV-5 V-protein. The alignment was performed using Seaview software.
(TIF)

Figure S14 Requirement of the CxCxxC motif, but not the DxR motifs, for DDB1 association and efficient STAT2 degradation by pM27. (**A**) Cells were infected with VACVs expressing indicated Flag-epitope tagged mutant proteins of pM27. Cells were lysed and subjected to anti-Flag immunoprecipitation. Precipitates were separated by SDS-PAGE and probed with Flag- and DDB1-specific antibodies. (**B**) NIH3T3 cells were infected with grading concentrations of VACVs (0.3, 3 and 9 PFU/cell). Cells were lysed and analyzed by western blotting using STAT2- and Flag-specific antibodies. An irrelevant background band served as intrinsic loading control. All panels are derived from the same blot and the same exposure.
(TIF)

Figure S15 pM27, but not HCMV pUL27, precipitates human DDB1. Human MRC-5 cells were infected (3 PFU/cell) with wt-VACV, M27-Flag-VACV or UL27-Flag-VACV. Cells were lysed and subjected to immunoprecipitation with anti-Flag antibody, an anti-V5 or an irrelevant control antibody. Precipitated proteins were separated by SDS-PAGE and analyzed by western blotting with the indicated antibodies.
(TIF)

Figure S16 siRNA-mediated ablation of DDB1. Primary MEF cells (passage 3) were transfected with 100 nM DDB1-specific siRNA (a luciferase-specific siRNA served as negative control), lysed 16, 24, 48 or 72 h post transfection and DDB1 amounts were determined by western blotting. β-actin served as protein loading control.
(TIF)

Figure S17 IFN-γ induces STAT2-containing, ISRE-DNA-element-binding, pM27-sensitive protein complexes. NIH3T3 cells were stimulated for 24 or 48 h with IFN-γ and infected (10 PFU/cell) with wt-MCMV, ΔM27-MCMV or left uninfected. Native protein lysates were prepared and an electromobility shift assay (EMSA) was performed using an ISRE probe as described previously [11]. STAT2-containing complexes were identified by a super-shift, using a STAT2-specific antibody (compare to mock lanes+IFN-γ). Comparable infection can be deduced from similar amounts of ‘CIF’ – a previously identified CMV-induced ISRE-binding complex [49].
(TIF)
