# Supplementary figures and images for: Identification of DNA-Damage DNA-Binding Protein 1 as a Conditional Essential Factor for Cytomegalovirus Replication in Interferon-γ-Stimulated Cells
Source: PLoS Pathog. 2011 Jun 16;7(6):e1002069. doi: 10.1371/journal.ppat.1002069 (PMC3116810; doi:10.1371/journal.ppat.1002069)

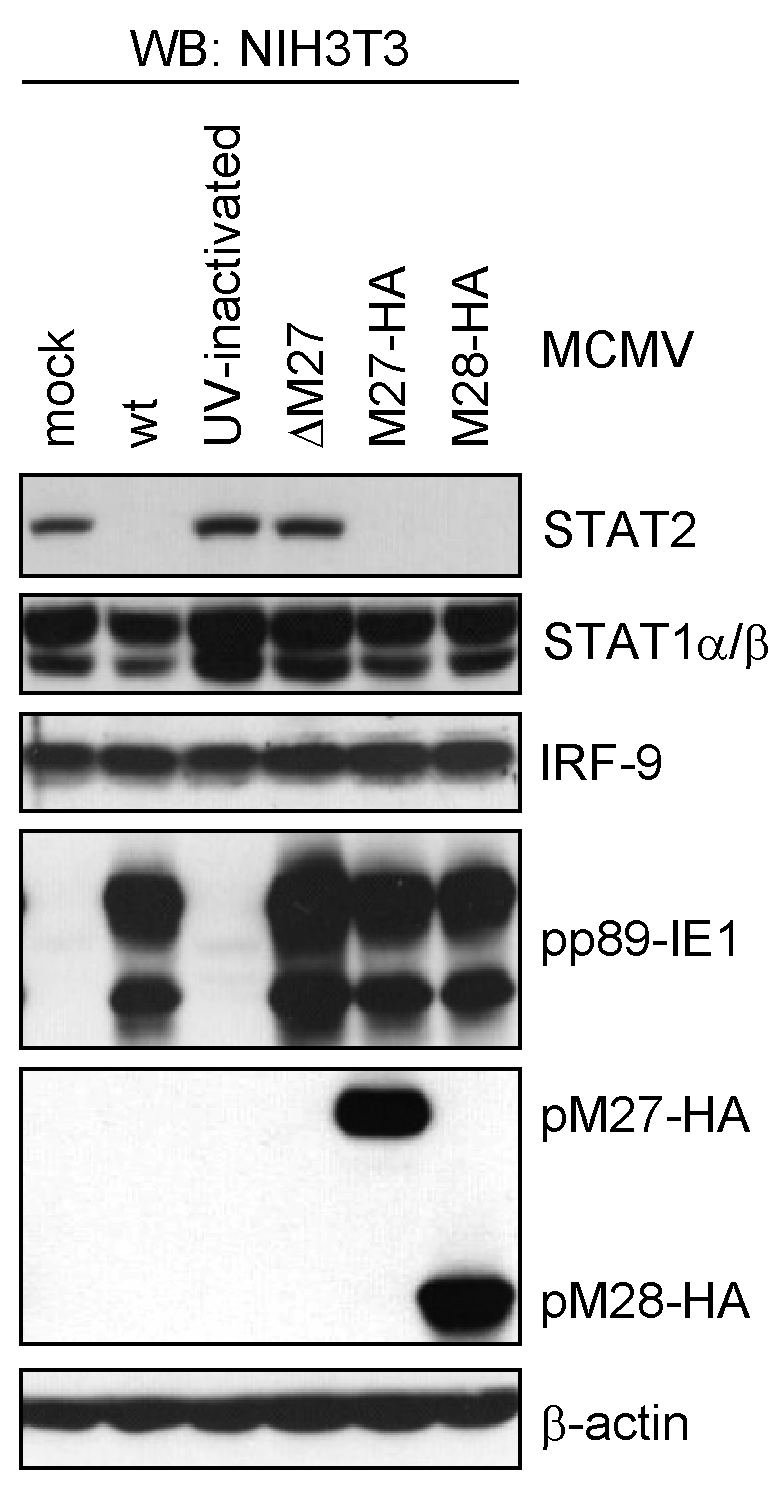

Supplement: Figure S1 — M27-HA-MCMV and M28-HA-MCMV, but not ΔM27-MCMV, reduces STAT2 amounts. NIH3T3 cells were infected with wt-MCMV, UV-irradiated MCMV, indicated MCMV mutants (5 PFU/cell, 48 h) or left uninfected. Cell lysates were adjusted and subsequently analyzed by western blotting with specific antibodies as indicated. All proteins were detected on one membrane in iterated rounds of detection. (TIF) [file ppat.1002069.s001.tif]

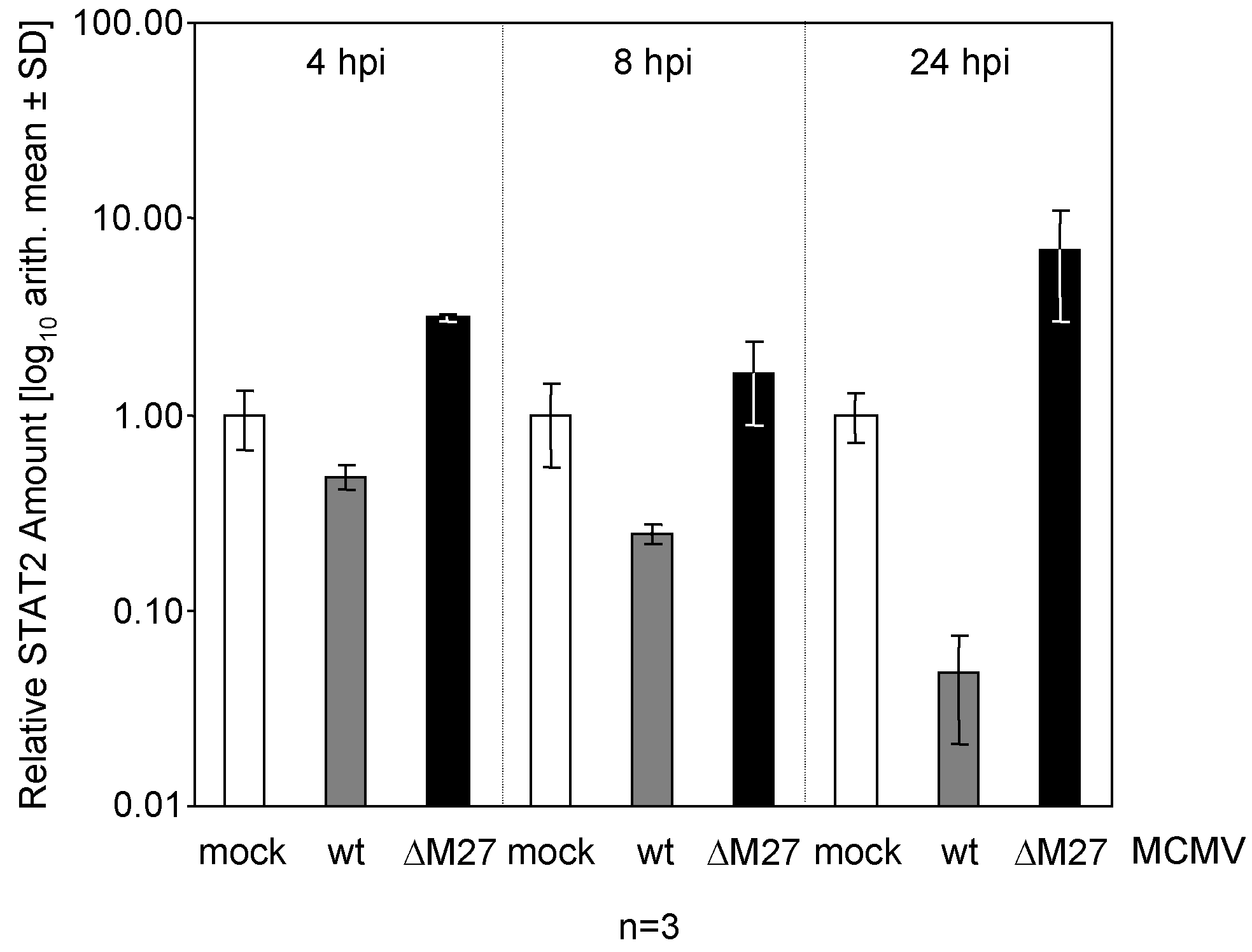

Supplement: Figure S2 — Quantification of STAT2 amounts in MCMV-infected cells. NIH3T3 cells were infected (10 PFU/cell) with ΔM27-MCMV, wt-MCMV or left uninfected. 4, 8 and 24 h post infection cells were lysed and the endogenous STAT2 amounts were determined by western blotting. Three western blots have been densitometrically quantified. The arithmetic mean ± SD of the relative STAT2 amount compared to mock-infected cells at the same timepoint is shown in a log10 scale. (TIF) [file ppat.1002069.s002.tif]

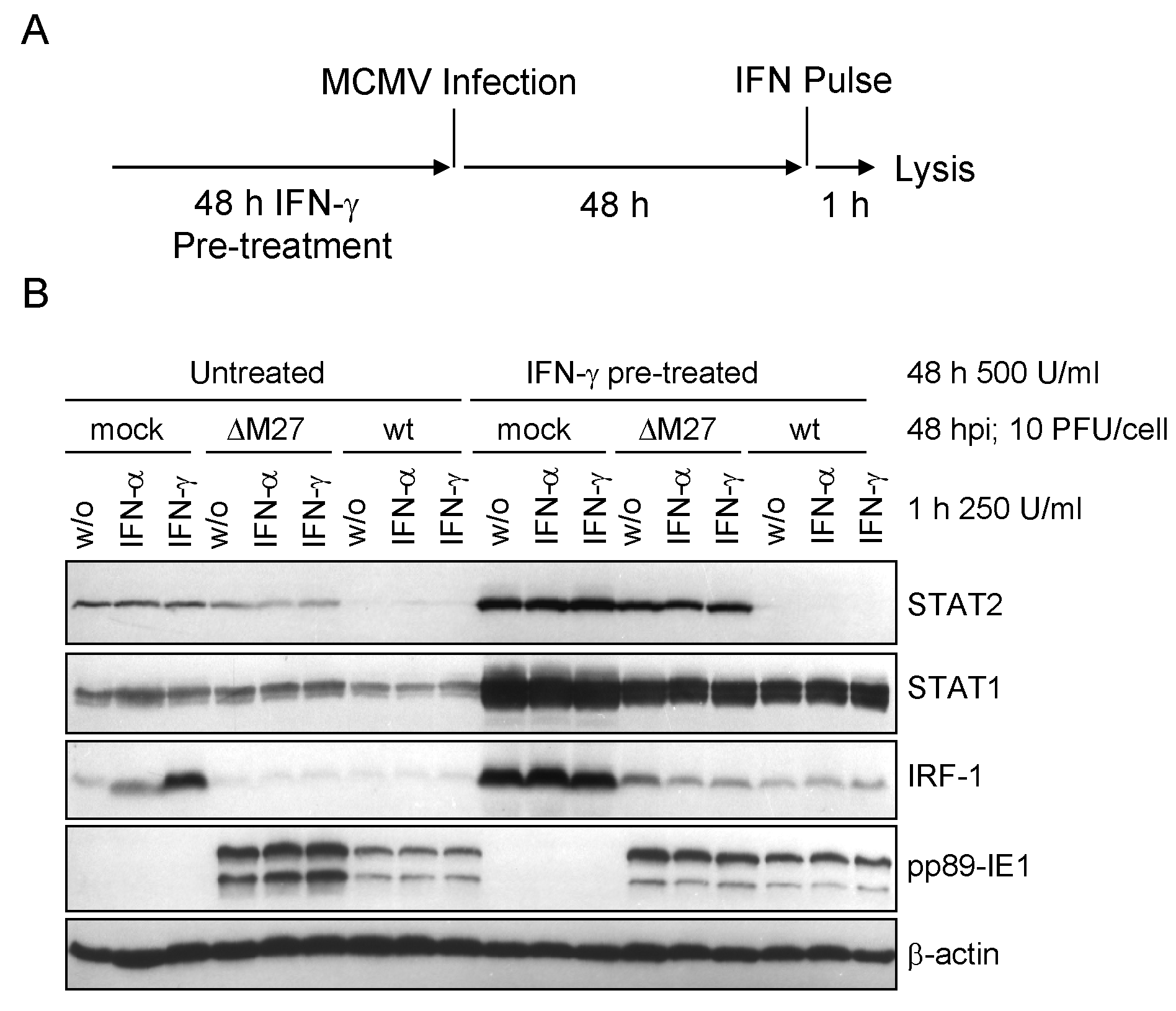

Supplement: Figure S3 — M27 reduces STAT2 even upon induction by IFN-γ (A) Schematic overview of the experimental setup. (B) Cells were incubated for 48 h with 500 U/ml IFN-γ prior to infection with wt-MCMV or ΔM27-MCMV (10 PFU/cell) for additional 48 h. Subsequently, cells were incubated for 1 h with 250 U/ml IFN-α or IFN-γ before lysis. Lysates were normalized and analyzed by SDS-PAGE and western blotting with the indicated antibodies. (TIF) [file ppat.1002069.s003.tif]

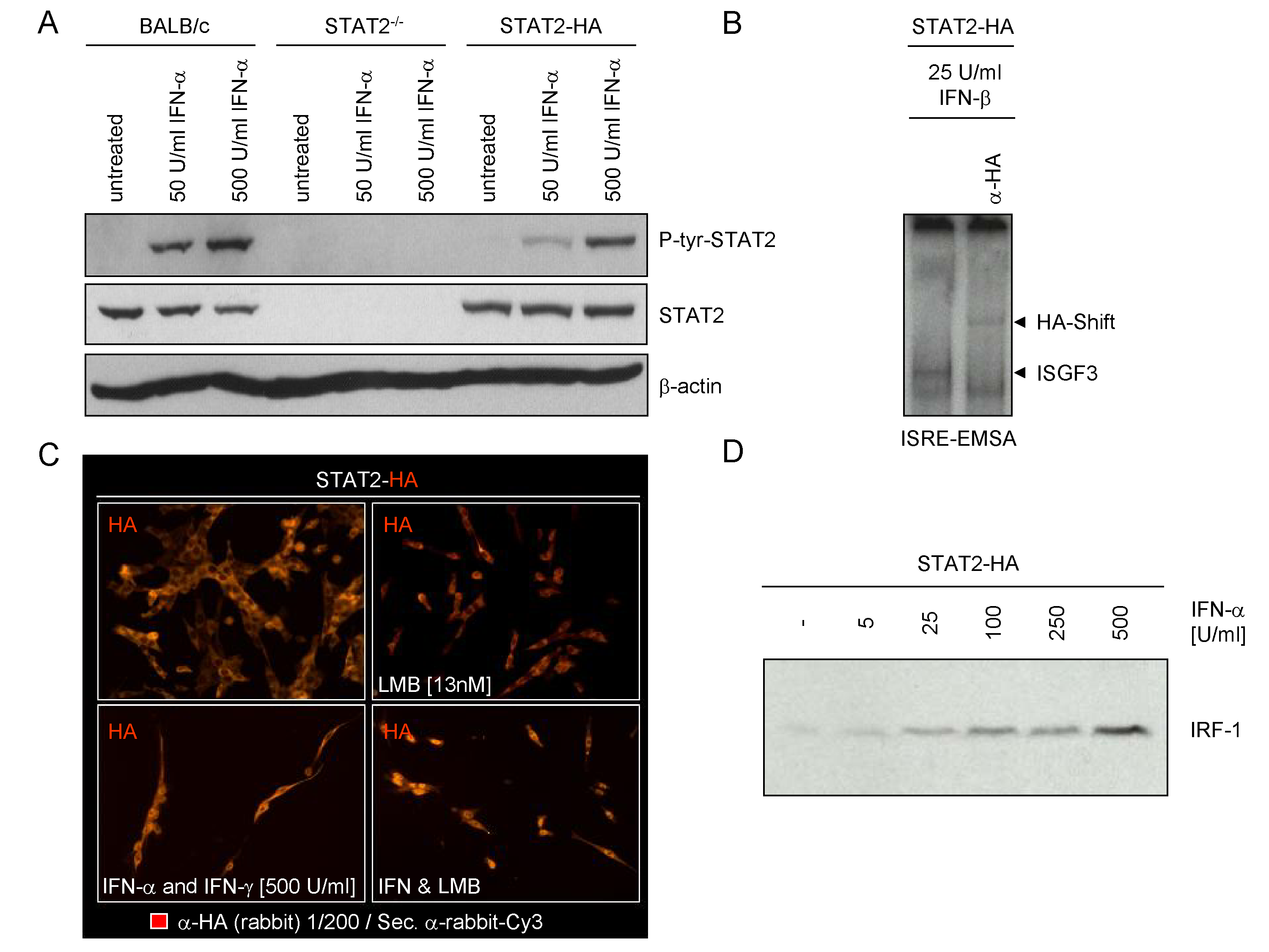

Supplement: Figure S4 — STAT2-HA transfectant express a functional STAT2. (A) STAT2-HA becomes tyrosine phosphorylated upon IFN stimulation. BALB/c MEF, STAT2−/− cells and STAT2-HA transfectants thereof were incubated with 50 or 500 U/ml IFN-α for 25 min. Cells were lysed and subjected to western blotting. (B) STAT2-HA forms ISGF3 complexes. Cells were stimulated with 25 U/ml IFN-β and native lysates were prepared and analyzed by electromobility shift analysis (EMSA) with a 32P-labelled ISRE probe as described before [11]. ISGF3 complexes were super-shifted upon addition of an HA-specific antibody. (C) STAT2-HA translocates in the nucleus upon IFN stimulation. STAT2-HA cells were incubated for 1 h with 500 U/ml IFN-α and IFN-γ. Additionally, leptomycin B (LMB) was administered as indicated to block the CRM-dependent protein export. Cells were fixed in 3% (v/v) PFA, permeabilized with 0.2% (v/v) Triton-X-100. STAT2-HA was detected with an HA-specific rabbit antibody and visualized with a Cy3-coupled secondary anti-rabbit antibody. (D) IRF-1 can be induced in STAT2-HA cells by IFN-α. STAT2-HA cells were incubated with grading concentrations of IFN-α and induced amounts of IRF-1 were detected by western blotting. (TIF) [file ppat.1002069.s004.tif]

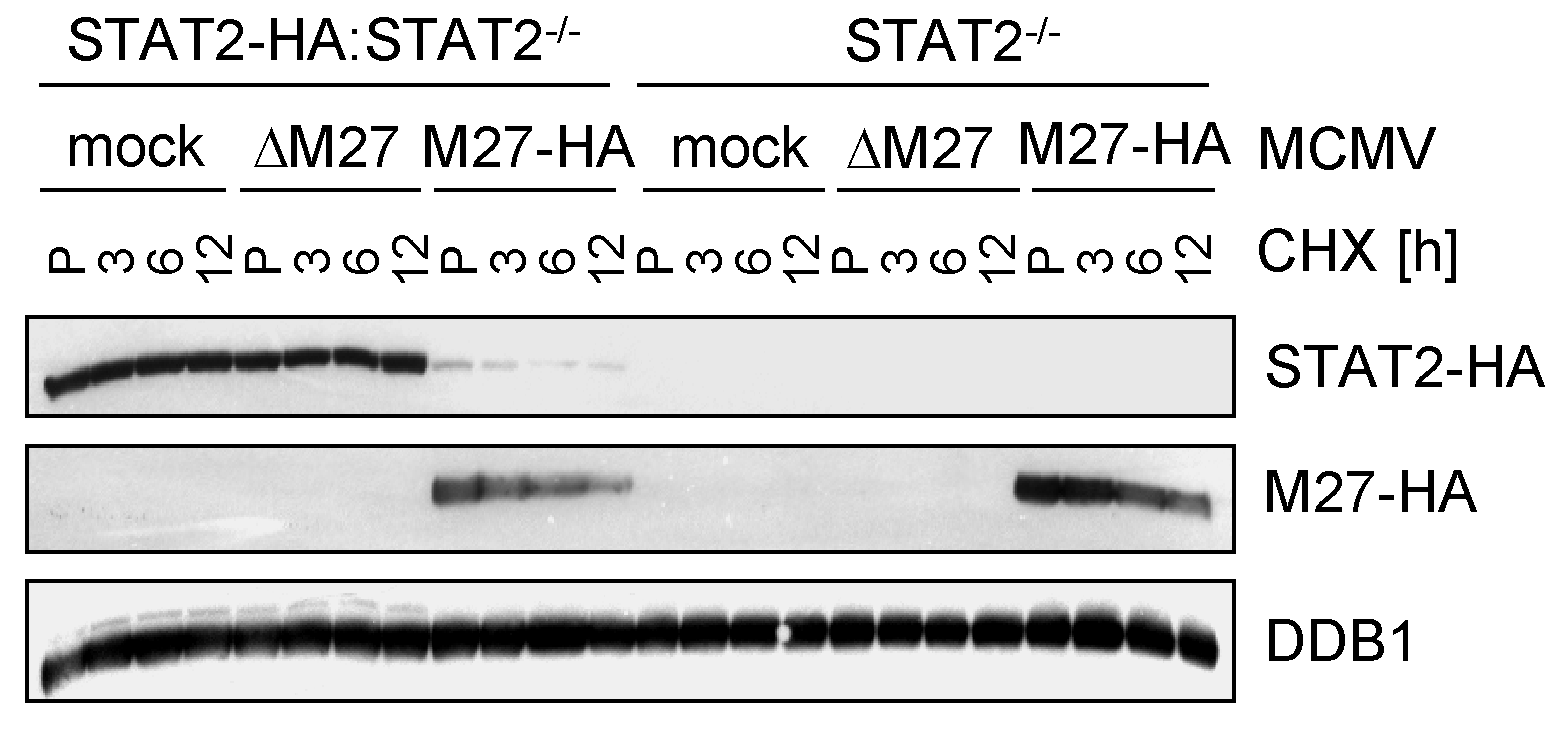

Supplement: Figure S5 — M27-HA-MCMV reduces STAT2-HA amounts whereas ΔM27-MCMV does not. STAT2-HA cells or the corresponding STAT2-deficient parental cells were infected (14 h; 5 PFU/cell) before CHX was added. Cells were directly lysed (Pulse) or at 3, 6 and 12 h post CHX addition and the lysates were analyzed by western blotting using the indicated antibodies. (TIF) [file ppat.1002069.s005.tif]

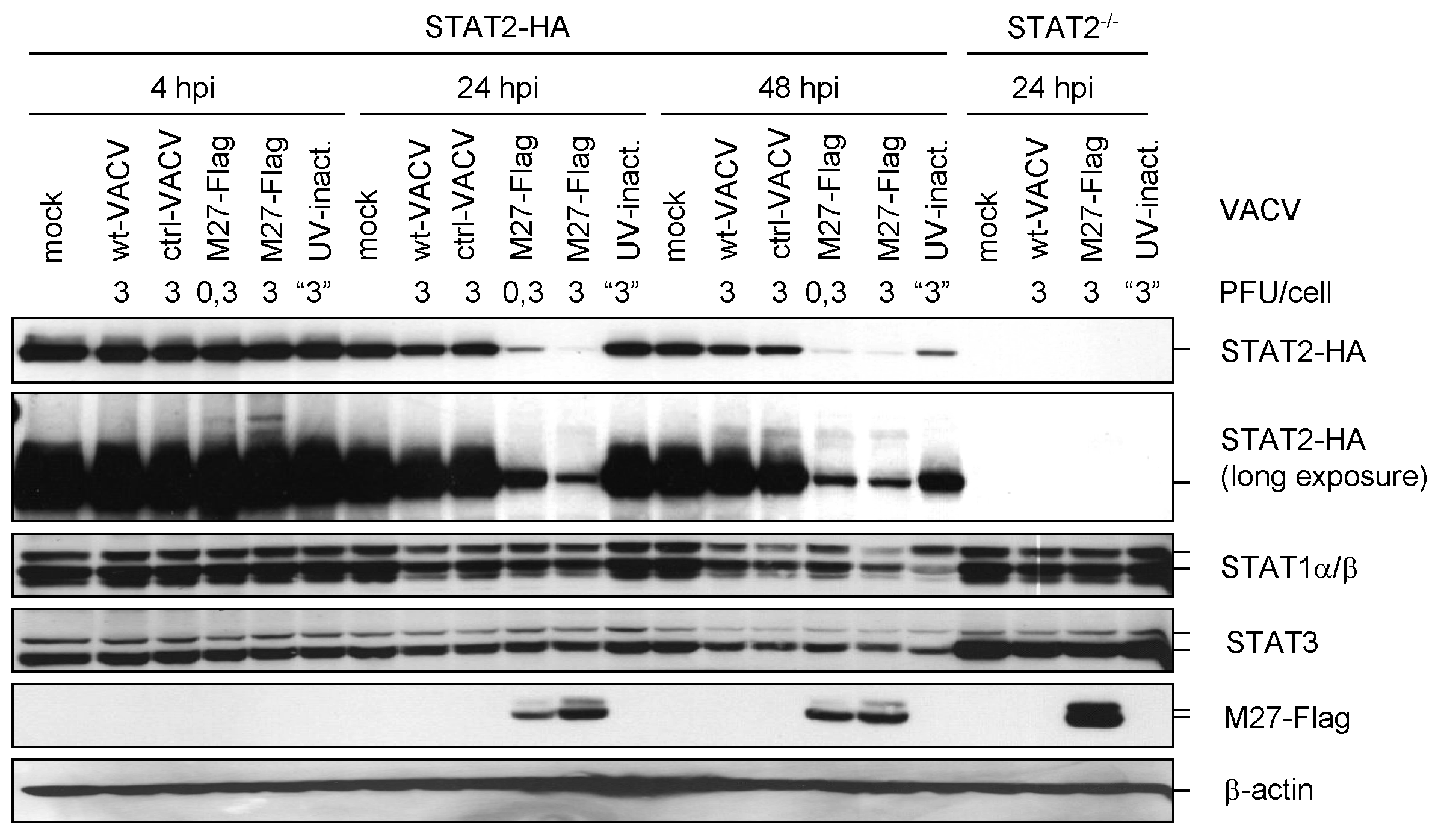

Supplement: Figure S6 — Loss of STAT2-HA upon infection with M27-Flag-VACV is time- and dose-dependent. A section of this experiment is shown in Figure 1B. STAT2-HA cells or the corresponding STAT2-deficient parental cells were infected with the indicated VACVs. Cells were lysed at indicated timepoints and subjected to western blotting. (TIF) [file ppat.1002069.s006.tif]

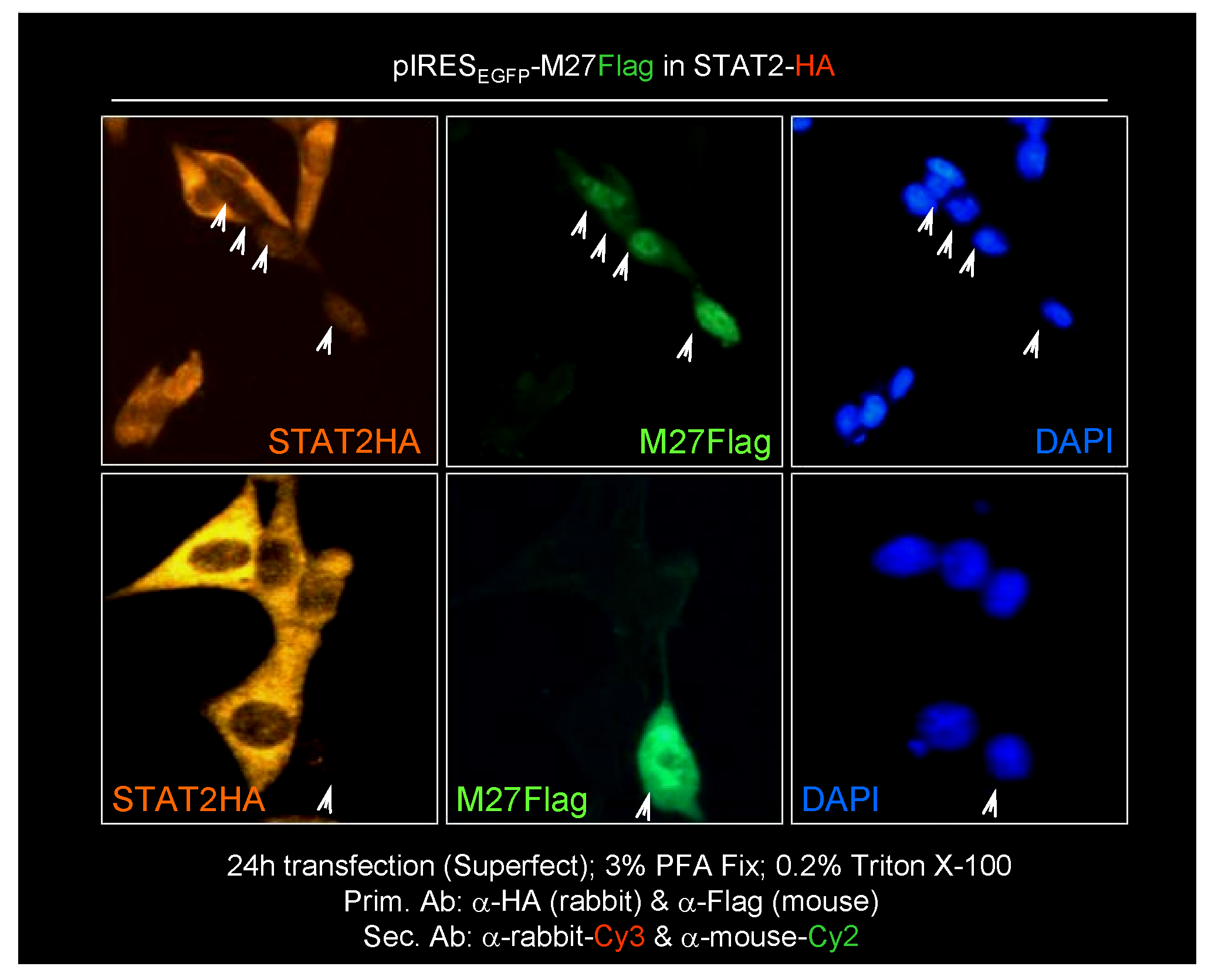

Supplement: Figure S7 — Transfection of M27-expression plasmids reduces STAT2 amounts on the level of an individual cell. STAT2-HA cells were transfected with a pM27-Flag expression plasmid (pIRESEGFP) using Superfect (Qiagen) transfection reagent, 24 h later fixed (3% [v/v] PFA), permabilized (0.2% [v/v] Triton-X-100) and stained with α-HA (rabbit), α-Flag (mouse) and secondary anti-rabbit (Cy3-coupled) or anti-mouse (Cy5-coupled) antibodies. IFM was done as previously described [14]. M27-transfected cells are indicated by white arrowheads. (TIF) [file ppat.1002069.s007.tif]

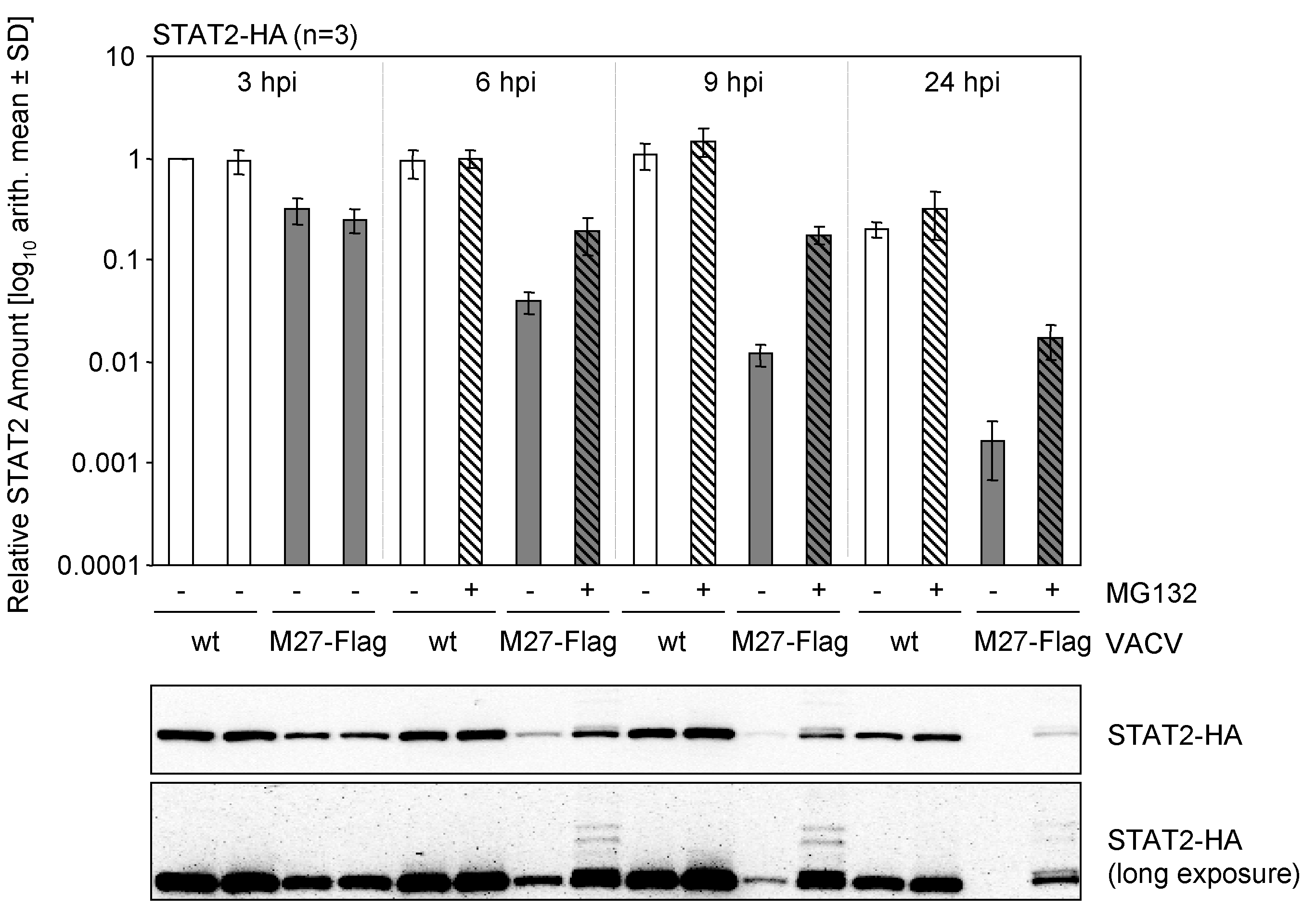

Supplement: Figure S8 — pM27-dependent reduction of the STAT2-HA half-life is MG132 sensitive. STAT2-HA cells were infected with wt-VACV, M27-Flag-VACV or left uninfected. 3 h post infection MG132 (20 µM) was added and cells were lysed at indicated time points. Three experiments were performed and densitometrically quantified. Shown is the arithmetic mean ± SD of the relative STAT2-HA amount compared to untreated wt-VACV infected cells. In the lower panel one representative western blot is shown. High molecular weight forms of STAT2 become apparent in the long exposure. (TIF) [file ppat.1002069.s008.tif]

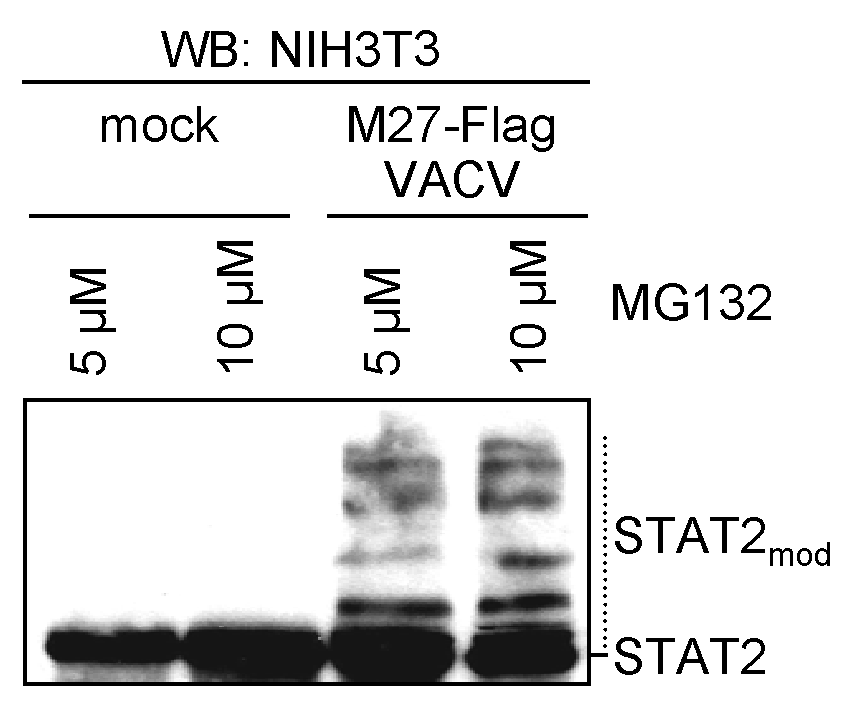

Supplement: Figure S9 — pM27 induces higher molecular weight forms of endogenous STAT2 upon incubation with MG132. NIH3T3 cells were infected with M27-Flag-VACV or left uninfected. Cells were incubated with 5 or 10 µg/ml MG132. Cells were lysed and lysates were subjected to western blotting with a STAT2-specific antibody. (TIF) [file ppat.1002069.s009.tif]

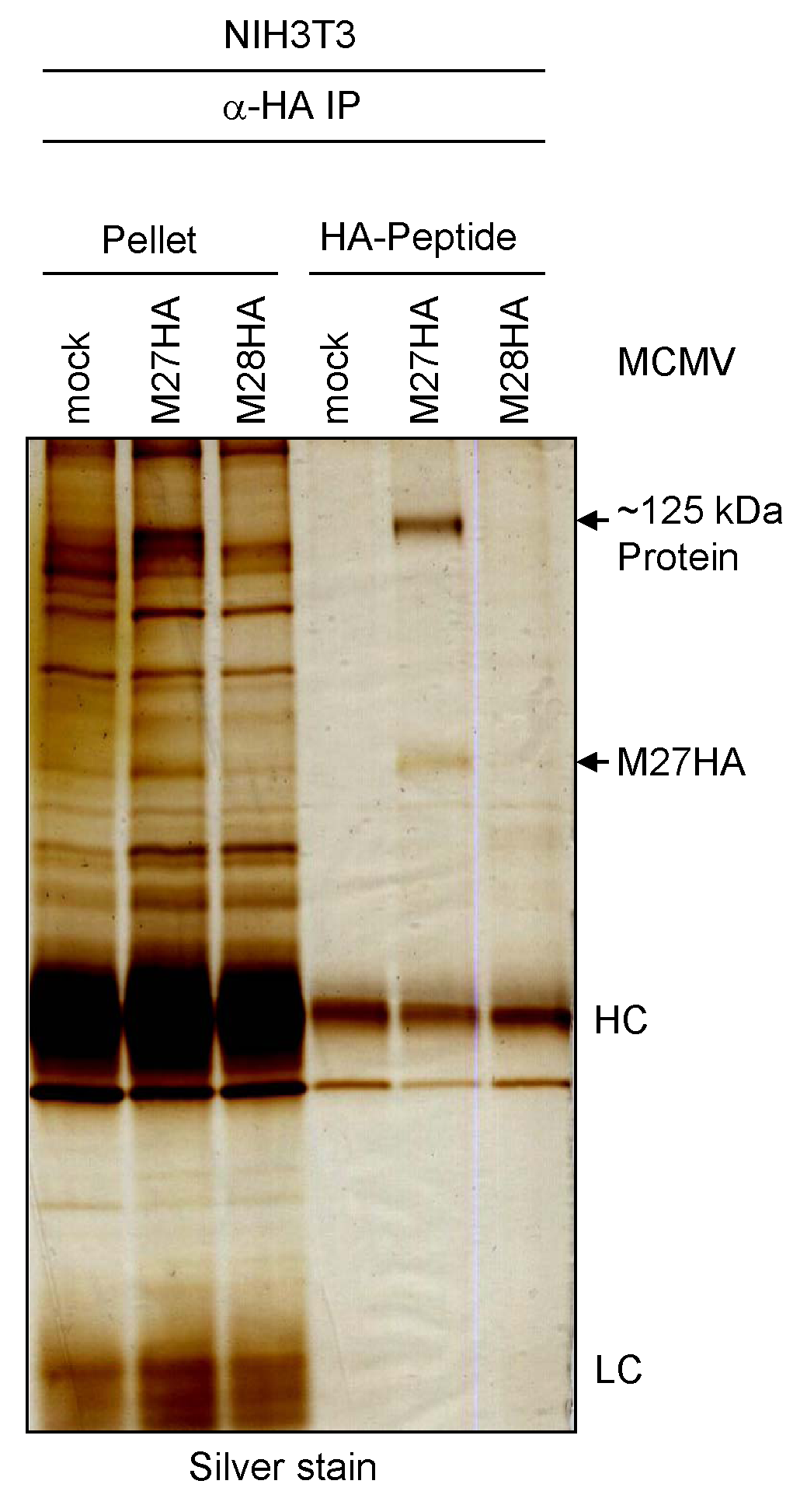

Supplement: Figure S10 — pM27-HA-DDB1 co-precipitation by HA-specific antibodies occurs epitope specific. NIH3T3 cells were infected with indicated mutants (24 h, 5 PFU/cell) or left uninfected. Cells were lysed and lysates were subjected to an immunoprecipitation with HA-specific antibody. Pellets of sepharose with retrieved immune complexes were incubated with an excess of HA-peptide (200 µg/ml in 10 mM Tris-HCl pH 8.0 for 25 min at 37°C). Supernatants were subjected to 8% SDS-PAGE and silver staining (right panel). Pellets were boiled in sample loading puffer and also analyzed by silver staining (left panel). LC, light chain, HC heavy chain of antibodies. (TIF) [file ppat.1002069.s010.tif]

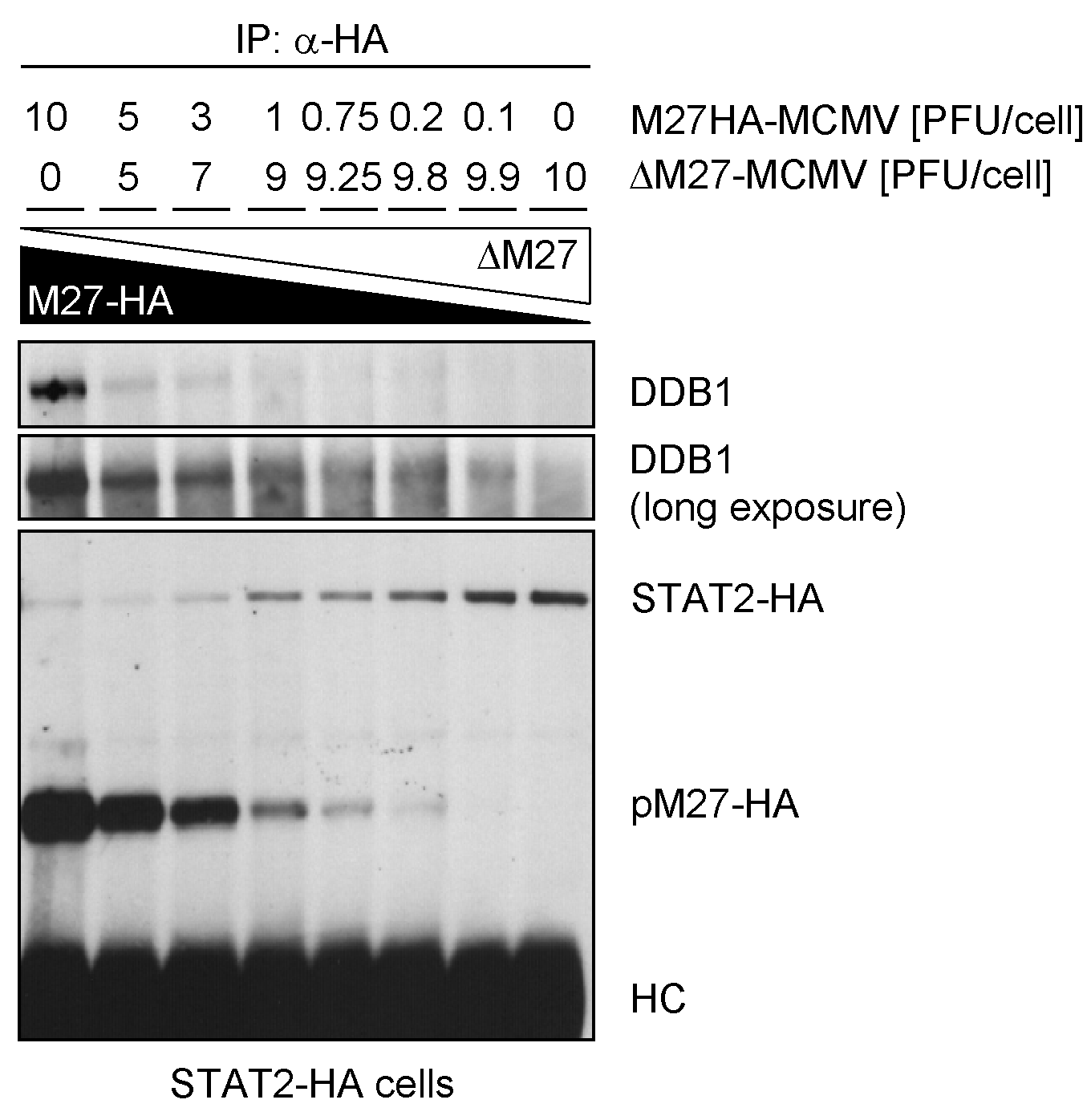

Supplement: Figure S11 — pM27-DDB1 co-precipitation and STAT2-degradation occur dose-dependently. STAT2-HA cells were infected with grading titers of M27-HA-MCMV. To ensure identical infection dose, ΔM27-MCMV was added to end up with a constant infectious dose of 10 PFU/cell. Cells were lysed and subjected to an anti-HA immunoprecipitation and subsequent western blotting with the indicated antibodies. (TIF) [file ppat.1002069.s011.tif]

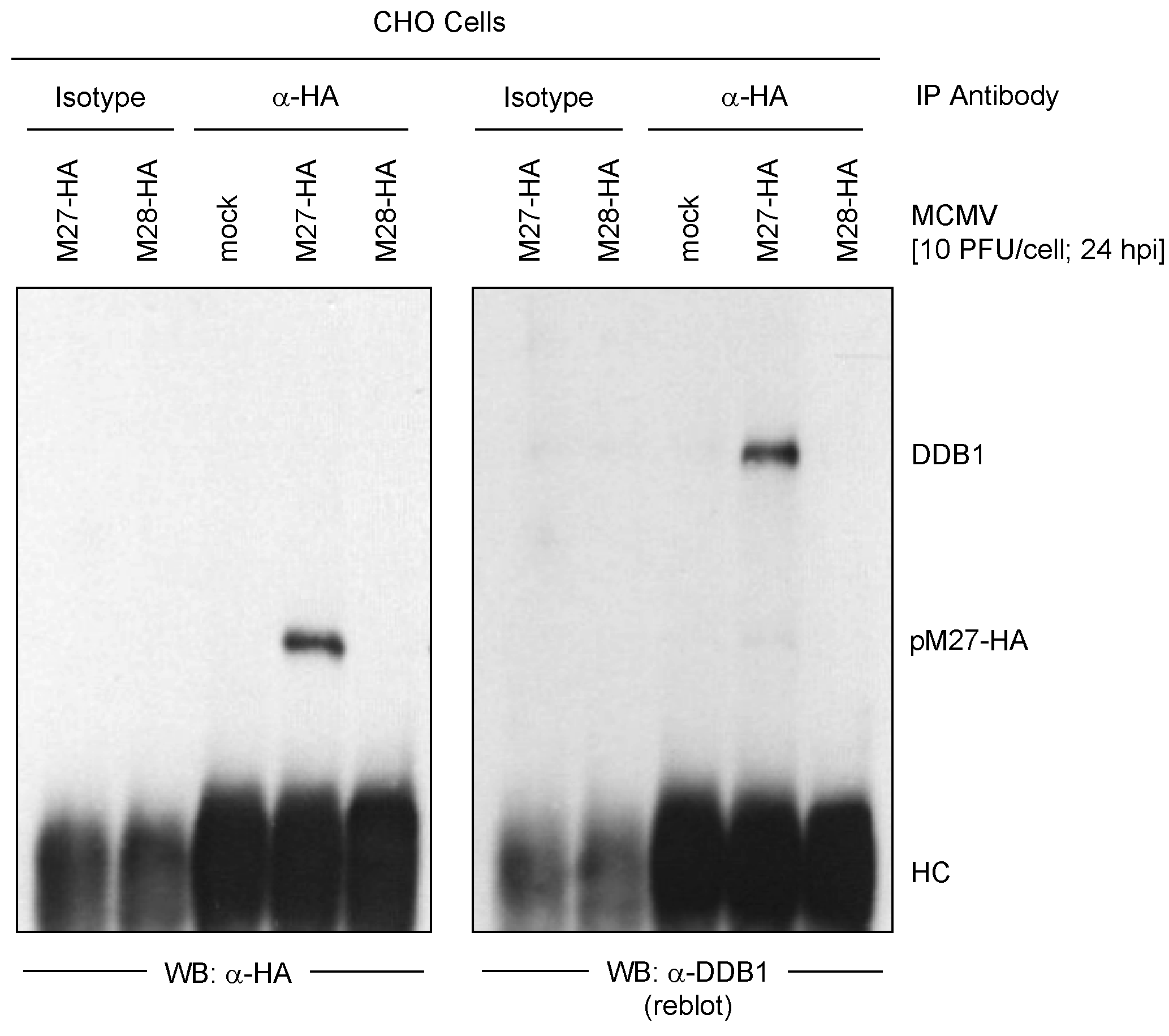

Supplement: Figure S12 — pM27 co-precipitates DDB1 in hamster CHO cells lacking DDB2. CHO cells were infected (24 h, 10 PFU/cell) with M27-HA-MCMV or M28-HA-MCMV or left uninfected. Cells were lysed and analyzed by immunoprecipitation with an HA-specific antibody. Immune complexes were analyzed by SDS-PAGE and western blotting with either an HA-specific or a DDB1-specific antibody. (TIF) [file ppat.1002069.s012.tif]

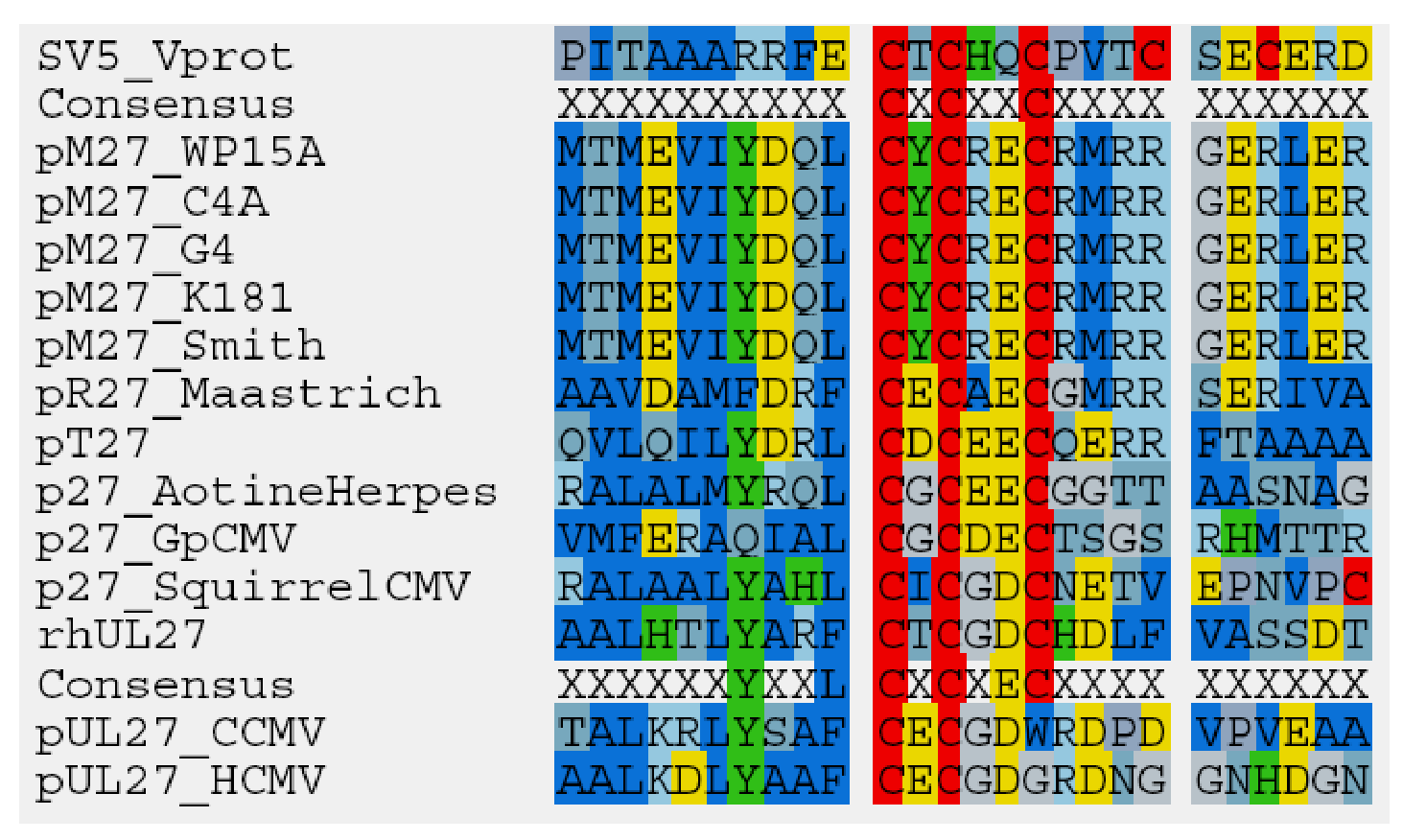

Supplement: Figure S13 — Alignment of CxCxxC motif in cytomegaloviruses. Sequences of indicated cytomegaloviral ‘27’ proteins were inspected for presence of CxCxxC motifs. Shown is an alignment of the 5 known MCMV pM27 sequences (WP15A, C4A, G4A, K181 and Smith) in comparison to the sequences derived from rat CMV (Maastricht), aotine CMV, tupaia CMV (pT27), guinea pig CMV (GpCMV), squirrel CMV, rhesus CMV (rhCMV), chimpanzee CMV (CCMV), human CMV (HCMV pUL27) and the SV-5 V-protein. The alignment was performed using Seaview software. (TIF) [file ppat.1002069.s013.tif]

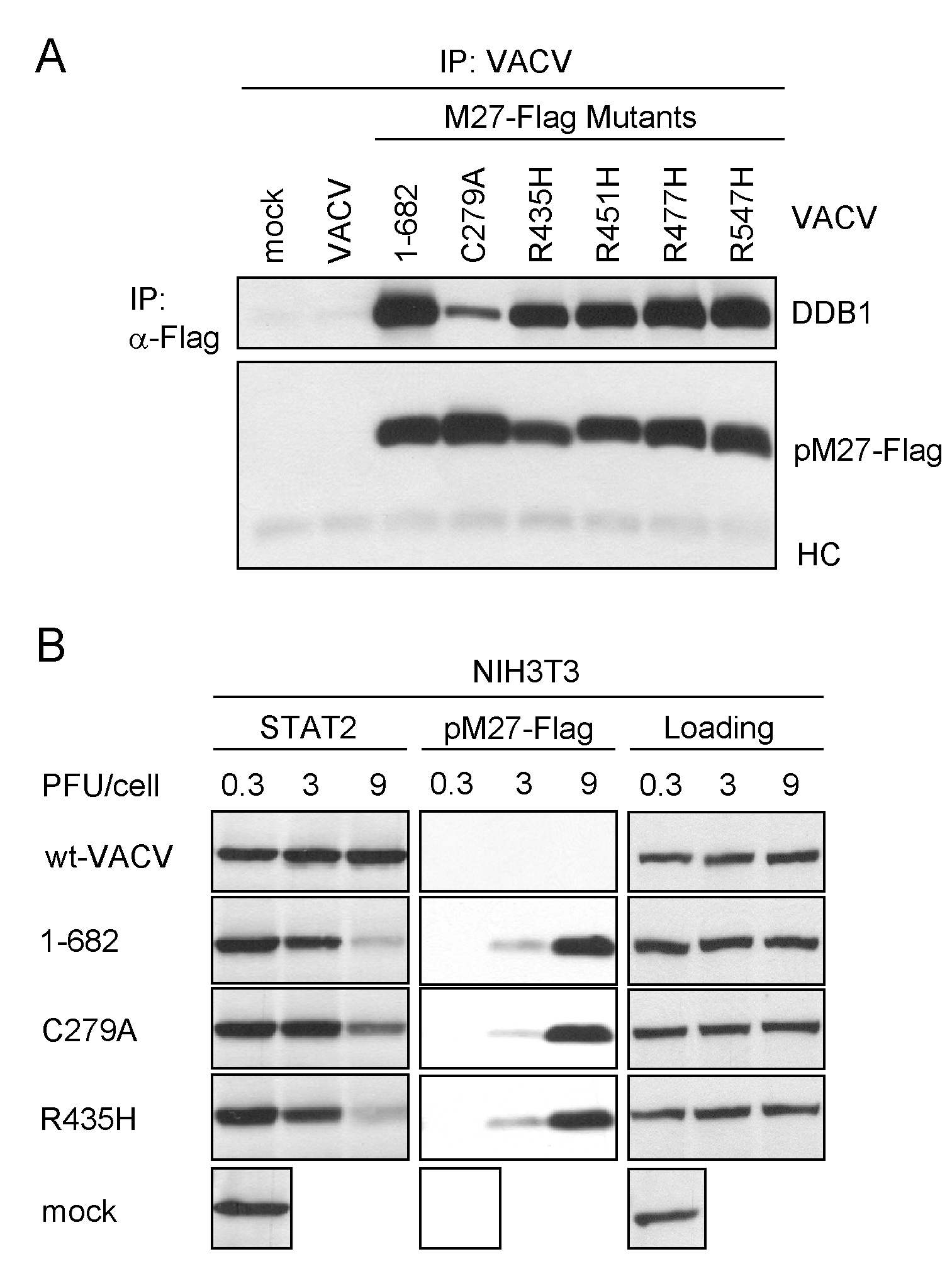

Supplement: Figure S14 — Requirement of the CxCxxC motif, but not the DxR motifs, for DDB1 association and efficient STAT2 degradation by pM27. (A) Cells were infected with VACVs expressing indicated Flag-epitope tagged mutant proteins of pM27. Cells were lysed and subjected to anti-Flag immunoprecipitation. Precipitates were separated by SDS-PAGE and probed with Flag- and DDB1-specific antibodies. (B) NIH3T3 cells were infected with grading concentrations of VACVs (0.3, 3 and 9 PFU/cell). Cells were lysed and analyzed by western blotting using STAT2- and Flag-specific antibodies. An irrelevant background band served as intrinsic loading control. All panels are derived from the same blot and the same exposure. (TIF) [file ppat.1002069.s014.tif]

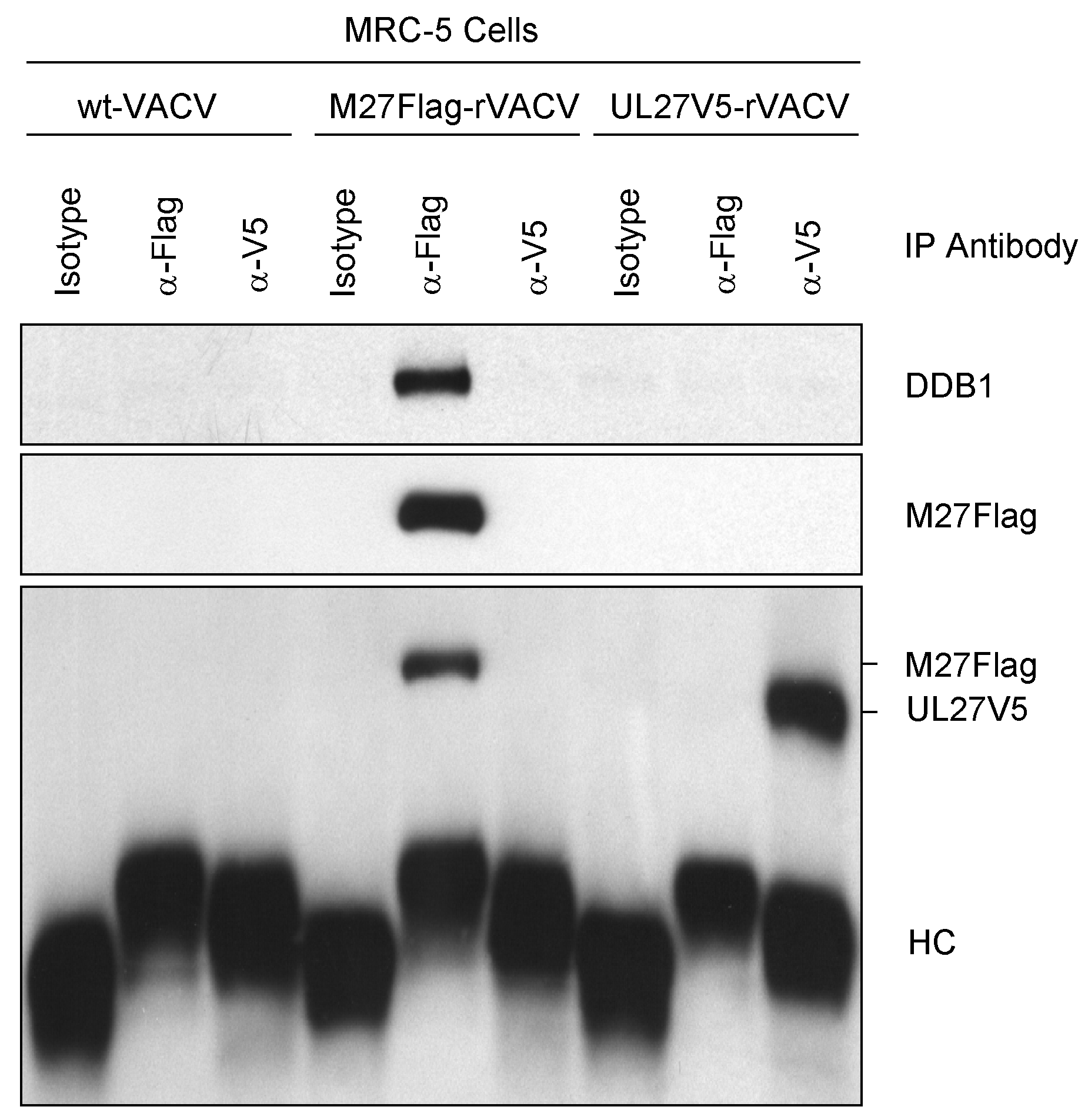

Supplement: Figure S15 — pM27, but not HCMV pUL27, precipitates human DDB1. Human MRC-5 cells were infected (3 PFU/cell) with wt-VACV, M27-Flag-VACV or UL27-Flag-VACV. Cells were lysed and subjected to immunoprecipitation with anti-Flag antibody, an anti-V5 or an irrelevant control antibody. Precipitated proteins were separated by SDS-PAGE and analyzed by western blotting with the indicated antibodies. (TIF) [file ppat.1002069.s015.tif]

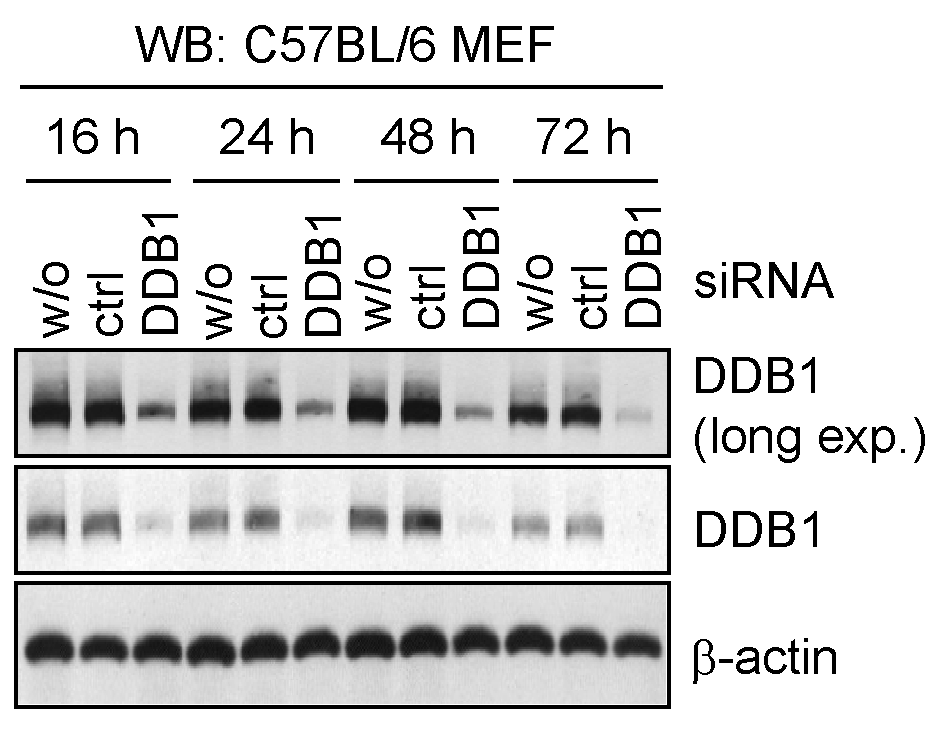

Supplement: Figure S16 — siRNA-mediated ablation of DDB1. Primary MEF cells (passage 3) were transfected with 100 nM DDB1-specific siRNA (a luciferase-specific siRNA served as negative control), lysed 16, 24, 48 or 72 h post transfection and DDB1 amounts were determined by western blotting. β-actin served as protein loading control. (TIF) [file ppat.1002069.s016.tif]

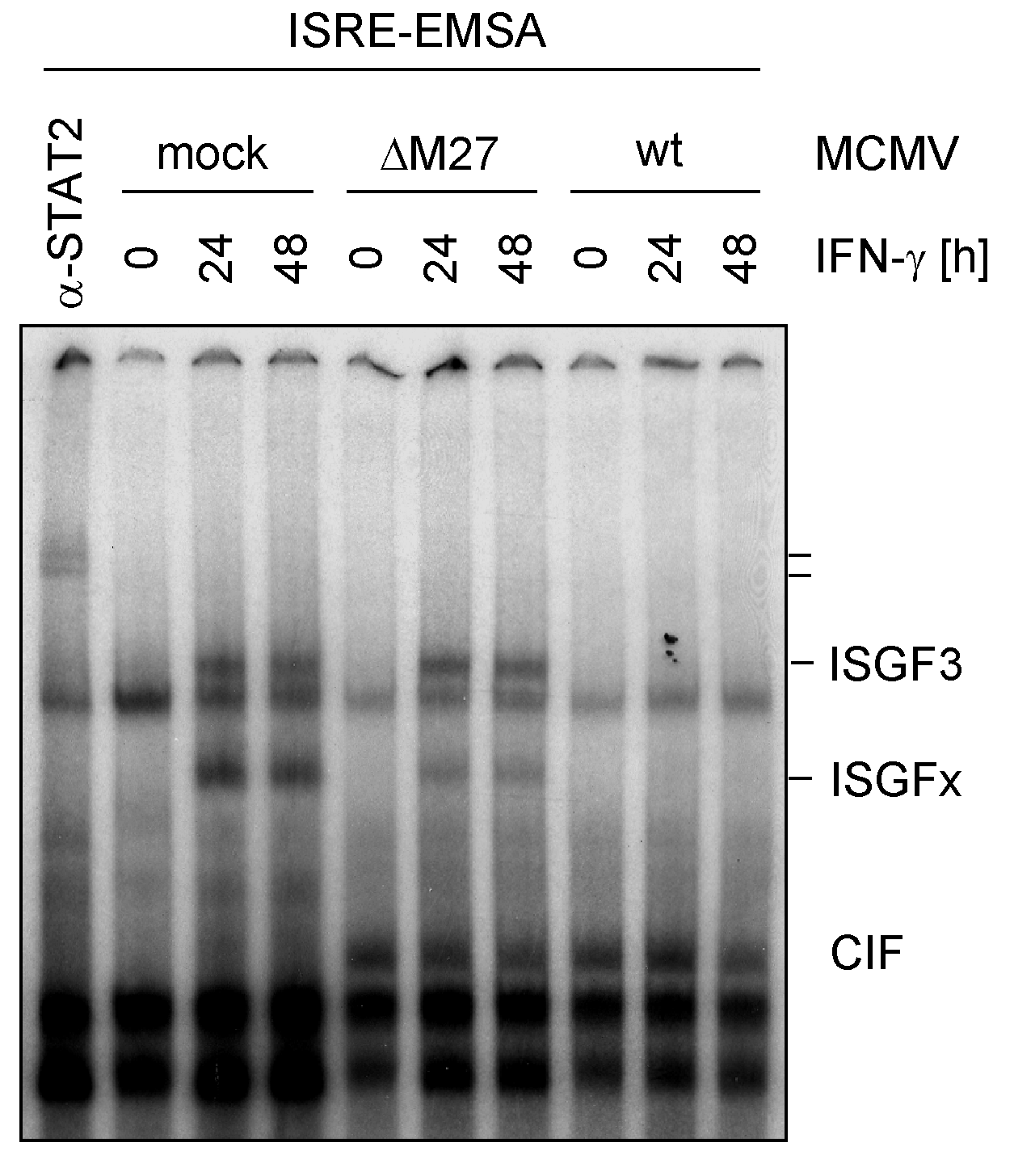

Supplement: Figure S17 — IFN-γ induces STAT2-containing, ISRE-DNA-element-binding, pM27-sensitive protein complexes. NIH3T3 cells were stimulated for 24 or 48 h with IFN-γ and infected (10 PFU/cell) with wt-MCMV, ΔM27-MCMV or left uninfected. Native protein lysates were prepared and an electromobility shift assay (EMSA) was performed using an ISRE probe as described previously [11]. STAT2-containing complexes were identified by a super-shift, using a STAT2-specific antibody (compare to mock lanes+IFN-γ). Comparable infection can be deduced from similar amounts of ‘CIF’ – a previously identified CMV-induced ISRE-binding complex [49]. (TIF) [file ppat.1002069.s017.tif]
